# Proteome Reprogramming and Acquired Stress Tolerance in Potato Cells Exposed to Acute or Stepwise Water Deficit

**DOI:** 10.1111/pce.15306

**Published:** 2024-12-05

**Authors:** Elisa Cappetta, Carmine Del Regno, Sara Ceccacci, Maria Chiara Monti, Lucio Spinelli, Marisa Conte, Chiara D'Anna, Mariaevelina Alfieri, Mariapia Vietri, Antonello Costa, Antonietta Leone, Alfredo Ambrosone

**Affiliations:** ^1^ Department of Pharmacy University of Salerno Fisciano Italy; ^2^ SAFE—School of Agricultural, Forest, Food, and Environmental Sciences University of Basilicata Potenza Italy; ^3^ Proteomics Platform Necker Université Paris Cité‐Structure Fédérative de Recherche Necker Paris France; ^4^ Department of Pharmacy University of Naples ‘Federico II’ Naples Italy; ^5^ Clinical Pathology, Santobono‐Pausilipon Children's Hospital, AORN Naples Italy; ^6^ National Research Council of Italy, Institute of Biosciences and BioResources, Research Division Portici (CNR‐IBBR) Portici Naples Italy

**Keywords:** potato (*Solanum tuberosum*), proteomics, regulatory networks, stress response mechanisms, water deficit tolerance

## Abstract

Water deficit negatively impacts crop productivity and quality. Plants face these challenges by adjusting biological processes and molecular functions according to the intensity and duration of the stress. The cultivated potato (*Solanum tuberosum*) is considered sensitive to water deficit, thus breeding efforts are needed to enhance its resilience. To capture novel functional information and gene regulatory networks, we carried out mass spectrometry‐based proteomics in potato cell suspensions exposed to abrupt or stepwise osmotic stresses. Both forms of stress triggered significant alterations in protein expression, though with divergent response mechanisms. Stress response pathways orchestrated by key proteins enrolled in primary and secondary metabolism, antioxidant processes, transcriptional and translational machinery and chromatin organization were found in adapted cells. Target metabolites and reactive oxygen species levels were quantified to associate functional outcomes with the proteome study. Remarkably, we also showed that adapted cells tolerate an array of diverse conditions, including anoxia, salt and heat stress. Finally, the expression patterns of genes encoding selected differentially expressed proteins were investigated in potato plants subjected to either drought or salt stress. Collectively, our findings reveal the complex cellular strategies of osmotic stress adaptation, identifying new fundamental genes that could enhance potato resilience.

## Summary statement

Plants respond to environmental stresses by modulating a complex network of molecular mechanisms according to the duration and intensity of the insult. Herein, we report the proteome reprogramming in potato cells under short and long‐term water deficit, providing unique molecular signatures of potato cell stress adaptation.

## 1 Introduction

Plant growth and productivity are markedly impaired by a spectrum of abiotic stressors, including, but not limited, to water deficit, soil salinity and high temperatures. These stresses are progressively intensifying in severity, duration and frequency due to the current climate change and pose an ever‐growing challenge to fulfill the increasing global demand for food (Benitez‐Alfonso et al. 2023).

Osmotic stress, a common feature in drought and high salinity conditions, significantly impacts essential biological processes like photosynthesis, protein biosynthesis and nutrient transport, which collectively culminate in pronounced decrements in yield. In this context, elucidating the mechanisms through which plants perceive and tolerate water deficit is fundamental for developing breeding tools aimed at mitigating the negative effects of water deficit on crop productivity.

In facing adverse external stimuli, plants initially perceive these environmental cues and subsequently orchestrate a cascade of cellular and molecular responses including modulations in the gene expression patterns, which, depending on the intensity and duration of the stress conditions, may confer tolerance (and ultimately survival) against stress, or conversely lead to plant death. Recent advancements in omics technologies, including genomics, transcriptomics, proteomics and metabolomics, have significantly boosted our understanding of plant responses to abiotic stressors (Cappetta et al. 2020; Sahoo et al. 2020; Basim et al. 2021). In particular, comprehensive transcriptomic analyses have enabled the delineation and characterization of an expanding repertoire of genes with functional and regulatory roles in the underlying mechanisms of the plant's abiotic stress response (Cao et al. 2016; Kamali and Singh 2023; Liu et al. 2023), which have revealed its complexity, involving changes in the protein metabolism, transcriptional and posttranscriptional regulation, ion homeostasis, detoxification, damage repair, signaling and stress perception (Xuan et al. 2022).

However, despite the enormous body of information gathered with the omics approaches, it is noteworthy that most of these studies have primarily focused on acute and severe stress conditions in crops and model plant species, while there is still a considerable gap in the comprehension of the genetic mechanisms underlying plant acclimation to prolonged and gradually increasing water deficit, which more closely reflects the field environment. Consequently, it is of paramount interest to discern between the transient responses to an acute stress constraint, more aimed at ensuring plant survival, from mechanisms facilitating the long‐term acquisition of tolerance. Understanding these mechanisms is essential for achieving a new cellular homeostasis, avoiding potential growth gap and ultimately preventing yield loss in crop plants. Novel knowledge of the gene networks involved in such gradual adaptation is crucial for designing advanced strategies aimed at developing climate‐resilient crops.

To address this gap, over the last decades, our research has focused on the cultivated potato, *Solanum tuberosum*, known for the vulnerability of many cultivars to water shortage. Predictions suggest that, by 2050, abiotic stresses could significantly reduce worldwide potato yields (Mańkowska, Zarzyńska, and Wasilewska‐Nascimento 2022; Sanwal et al. 2022; Demirel 2023). As the foremost non‐grain food crop, understanding the molecular mechanisms governing potato response and acclimation to water deficit is imperative, especially considering the increasing frequency and intensity of osmotic stresses. To this aim, we have developed an improved and robust in vitro model for studying potato cell responses to both abrupt or gradual water shortage, mimicked by the addition of polyethylene glycol (PEG) to the culture medium.

So far, the results obtained using this model have been very informative and have elucidated key cellular and molecular responses essential for maintaining homeostasis under stress, including the restoration of normal protein synthesis, accumulation of proline and polyamines and modifications in membrane composition and fluidity in response to gradual water deficit (Leone et al. 1994a, 1994b, 1996; Scaramagli et al. 2000). We have shown that short‐term exposure to severe water stress mainly activates genes associated with stress perception, signaling and the prevention or repair of cellular damage (Leone et al. 1994a, 1994b; Ambrosone et al. 2011, 2017). In addition, we demonstrated that selected genes specifically induced in adapted cells were able to confer stress tolerance *in planta* through comprehensive functional analyses, confirming the validity of this experimental approach (Ambrosone et al. 2015; Punzo et al. 2020).

In this study, we utilize the potato cell culture system to explore global proteome changes by high‐resolution mass‐spectrometry, going beyond our previous transcriptomic analyses (Ambrosone et al. 2017). The expanded proteomic analyses were carried out with the final aim of capturing novel functional information, interaction networks, posttranscriptional and posttranslational regulatory patterns that directly participate in water stress response and acquisition of tolerance.

Herein, proteomics analyses identified a few hundred differentially expressed proteins (DEPs) in the different potato cell populations. Through a detailed investigation employing Gene Ontology (GO), KEGG pathway and MapMan analyses, we elucidated the divergent cellular processes and molecular mechanisms underlying the potato cell response to abrupt versus gradual and long‐term osmotic stress. Interestingly, an extensive proteome remodeling was observed in gradually adapted potato cells, establishing a new cellular homeostasis able to support potato cell viability and conferring also cross‐tolerance to low oxygen, salt and heat stress. Finally, expression patterns of selected water stress‐responsive genes encoding key DEPs identified in adapted cells were also confirmed in potato plants exposed to drought and salinity, allowing the identification of promising targets in the regulation of the plant's adaptive responses to osmotic stress.

In conclusion, we demonstrated that potato cells respond to water deficit according to the intensity and duration of water‐limiting conditions, providing unique molecular signatures of potato cell stress adaptation. These insights offer valuable information for selecting tolerant potato genotypes and contribute to delineating a broader plant strategy for mitigating the negative effects of climate change on potato yield.

## 2 Materials and Methods

### 2.1 Potato Cell Culture and Osmotic Stress

Potato cell cultures were obtained from leaf callus of potato plants (cv. Desirée) as described by Leone et al. (1994a) and Tavazza, Ordás, and Ancora (1988). Control cells were maintained for 3 months in a modified Murashige and Skoog (MS) medium on a rotary shaker at 28°C in the dark, subcultured every 7 days in 50 mL fresh medium and monitored according to the methods described in Leone et al. (1994a). After stabilizing the cell cultures, PEG‐shocked cells were obtained by directly exposing 48 h‐ sub‐cultured cells to media containing 10% or 20% PEG for 24 h to obtain moderate and severe shock conditions, respectively. Control cells, used for comparison with the stressed conditions, were treated exactly like the two stressed samples but without the addition of PEG. Both sets of cells were then prepared for proteomic analysis. For gradual adaptation, water stress was imposed by a stepwise addition of PEG (average mol wt 8000; P5413, Sigma‐Aldrich) to the medium, beginning at 0% and escalating through 5%, 10%, 15% to 20% w/v PEG, corresponding to osmotic potentials of approximately –0.5, –1.0, –1.4, –1.85 and –2.3 MPa, respectively. To induce adaptation, the cells were maintained at least for two sub‐culture cycles at PEG concentration before transfer to the next PEG concentration. After completing these adaptation cycles, each cell population was cultured for an additional 4 weeks in their respective adaptation media as a final stabilization step. For protein extraction and proteomic analysis, the 10% and 20% PEG adapted and their corresponding control cells were harvested 72 h after sub‐culturing in their respective adaptation and control media.

### 2.2 Protein Extraction, Preparation and LC‐MS/MS Analyses

To prepare total protein extracts, the different liquid cell populations were filtered through filter paper, washed thoroughly with MS medium (pH = 5.8) to remove the PEG residues and immediately frozen in liquid nitrogen.

Cells (150 mg) were thawed in extraction buffer containing 100 mM NaH_2_PO_4_, 10 mM Tris (pH=8), 2 M urea and 1 mM PMSF, mixed and then centrifugated (13 000 rpm, 10 min, 4°C) to collect supernatants. Protein concentration of the six samples was determined by Bradford analysis (Bio‐Rad) and the same quantity of each protein sample (15 µg) was loaded on a 12% polyacrylamide gel, using a short electrophoresis run in the stacking part of the gel without a real protein separation and it was stained with a Coomassie solution. Following, the single broad band present in each lane was excised and digested by trypsin as reported by Shevchenko et al. (2006). The obtained peptide mixtures were dried under vacuum and dissolved in 10% formic acid (FA) for the subsequent nano LC‐MS/MS analysis. Peptides were analyzed on an Orbitrap QExactive Classic Mass Spectrometer (ThermoFisher Scientific) coupled to an UltiMate 3000 Ultrahigh‐Pressure Liquid Chromatography (UHPLC) system (ThermoFisher Scientific), equipped with an EASY‐Spray PepMAP RSLC C18 column (3 μm, 100 Å, 75 μm × 50 cm, ThermoFisher Scientific) at a flow rate of 300 nL/min with the following gradient: 1 min at 3% B, 1 to 100 min to 38% B, 100 to 101 min to 80% B, then held at 80% B for 10 min, and re‐equilibrated for 8 min at 3% B (A: 95% H_2_O, 5% CH_3_CN, 0.1% AcOH; B: 95% CH_3_CN, 5% H_2_O, 0.1% AcOH). The mass spectrometer was operated in data‐dependent acquisition mode. Full‐scan MS spectra were acquired with the scan range 375 − 1500 *m/z*, a full‐scan automatic gain control (AGC) target 3e6 at 70 000 resolution, and a maximum injection time of 50 ms. MS2 spectra were generated for up to eight precursors (normalized collision energy of 28%), and the fragment ions were acquired at a resolution of 17 500 with an AGC target of 1e5 and a maximum injection time of 80 ms. Protein identification and label‐free quantification were then achieved through Proteome Discoverer (version 2.4.1.15). The analysis was performed by Sequest against a reviewed *Solanum tuberosum* database (SwissProt, February 2022, 53 106 entries) with the following parameters: trypsin digestion; maximum of two missed cleavages; cysteine carboxyamidomethylation as fixed modification. Mass tolerances were 10 ppm for MS1 and 0.02 Da for MS/MS. Label‐free quantification was achieved by using unique and razor peptides for protein abundance calculation, Proteome Discoverer's standard normalization method a pairwise ratio‐based approach was used to evaluate the treated versus control peptides and protein abundance. Three biological replicates for each sample were performed. Only proteins with abundance ratio ≤ 0.65 (downregulated proteins) and ≥ 1.5 (upregulated proteins) and FDR‐corrected *p*‐value less than 0.05 were considered significant. A “2 out of 3” filtering rule has been implemented for an appropriate deletion of those proteins that could not be accurately measured because of missing values.

### 2.3 Bioinformatic Analyses

ShinyGO V0.8 was used for determining the GO terms of DEPs identified and describing the associated biological process, cellular component and molecular function by selecting *Solanum tuberosum* (SolTub_3.0) as a reference species (Ge, Jung, and Yao 2020). An FDR cut‐off of 0.05 was used to identify statistically significant results. The KEGG tool and MapMan software (version 3.5.1 R2) were used to visualize the involvement of DEPs in the pathways and annotate functional MapMan bin codes (BINs). The protein−protein interaction (PPI) profile of the 24 upregulated proteins identified belonging to the BIN 19 was obtained from the STRING webserver (https://string-db.org/). A high confidence (0.4) was selected as the minimum required interaction score. To cluster the most similar nodes of the network into an easily distinguishable function‐based classification, we employed the Markov Cluster Algorithm (MCL) with the infiltration factor setting at 3.

For promoter analysis of each selected gene, 2000 nucleotides upstream of the translation initiation codon were retrieved using Biomart in Ensembl Plants (https://plants.ensembl.org/index.html) and then analyzed using the PlantCARE tool (Lescot 2002).

### 2.4 LC‐ESI‐MS Quantification of Phenylpropanoids and Organic Acids

Cell samples from control, 20% PEG stress and 20% PEG adapted conditions were collected and lyophilized. Each sample (100 mg) was diluted in 2.5 mL of a 25:75 H_2_O:EtOH mixture, vortexed, sonicated for 45 min at 45°C and 35 kHz. The extracts were centrifuged and the supernatants were dried (Tungmunnithum et al. 2019).

For the analysis of phenylpropanoids, the stock solutions of five phenylpropanoids, namely t‐cinnamic acid at M‐H 163.0401 u.m.a, ferulic acid at M‐H 193.0506 u.m.a, sinapic acid at M‐H of 223.0612 u.m.a, caffeic acid at M‐H of 179.0350 u.m.a and chlorogenic acid at M‐H of 353.0878 u.m.a were prepared separately in methanol at a concentration of 10 mM. All stock solutions were stored at –20°C. Calibration standards were prepared by combining appropriate volumes of each stock solution and methanol. The calibration range was from 10 nM to 10 μM of each molecule in the final solution. The extracted cell samples were diluted in 50:50 H_2_O:MeOH mixture.

For the analysis of succinic acid and pyruvic acid, a derivatisation has been achieved using ECD (1‐ethyl‐3‐(3‐dimethylaminopropyl)carbodiimide hydrochloride) and phenylhydrazine hydrochloride. First, EDC and phenylhydrazine were added to the stock solution mixtures of 1–100 μM of short‐chain fatty acids at 30 and 50 mM, respectively, and the reaction occurred for 60 min at 37°C. The cell extracts were treated in the same way. The reaction was blocked using 1% FA. The mono‐ and di‐modified adducts were monitored as follows: succinic acid MH+ of 299.1503 u.m.a and pyruvic acid MH+ of 269.1397 u.m.a.

LC‐MS runs were carried out on an UHPLC‐MS system Orbitrap Tribrid Mass Spectrometer from ThermoFisher Scientific equipped with Vanquish Flex LC and AutoSampler systems.

For the analysis of phenylpropanoids, the mixture was separated on a Luna Omega Polar C18 1.6 μm from Phenomenex (150 mm × 2.1 mm) at 0.4 mL/min using an acetonitrile–aqueous gradient. Mobile phase A was water at 0.1% acetic acid, mobile phase B was acetonitrile at 0.1% acetic acid. The gradient started at 5% B, kept constant for 2 min and increased to 50% B in 8 min followed by washing and equilibration. ESI was performed in negative ion mode in a scan range of 100–500 *m/z* and the ion source temperature was set at 350°C. The source parameters were the following: negative ion voltage at 2500, sheath gas at 50 arb. units, aux gas at 10 arb. units, sweep gas at 1 arb. units, ion transfer tube temperature at 325°C. The orbitrap parameters were the following: orbitrap resolution 30.000, RF lens at 30%; normalized AGC target at 25%, maximum injection time 54 ms.

For the analysis of organic acids, the mixture was separated on a Luna Omega Polar C18 1.6 μm from Phenomenex (150 mm × 2.1 mm) at 0.35 mL/min using an acetonitrile–aqueous gradient and the column temperature was set at 50°C. Mobile phase A was water at 0.1% FA, mobile phase B was acetonitrile: H_2_O 80:20 at 0.1% FA. The gradient started at 1% B, kept constant for 2 min and increased to 60% B in 8 min followed by washing and equilibration. ESI was performed in positive ion mode in a scan range of 50–450 *m/z* and the ion source temperature was set at 350°C. The source parameters were the following: positive ion voltage at 3500, sheath gas at 50 arb. units, aux gas at 10 arb. units, sweep gas at 1 arb. units, ion transfer tube temperature at 325°C. The orbitrap parameters were the following: orbitrap resolution 30.000, RF lens at 30%; normalized AGC target standard, maximum injection time auto. The quantification was achieved using Quan Browser software from ThermoFisher Scientific.

### 2.5 Measurement of Intracellular Reactive Oxygen Species (ROS)

The intracellular generation of ROS under control, 20% PEG stress and 20% PEG adapted conditions was determined by detecting the fluorescence of dichlorofluorescein (DCF), the product of oxidation of the oxidant‐sensitive dye 6‐carboxy‐2′,7′‐dichlorodihydrofluorescein diacetate (DCFH‐DA, Sigma Aldrich) (Gao et al. 2008).

For the experiment, each cell sample (100 mg of control, 20% PEG‐shocked and 20% PEG‐adapted) was incubated with 1 μM DCFH‐DA in 1 mL of liquid MS medium (pH = 5.8) for 20 min on a rotary shaker. As a positive control, 100 mg of cells were preincubated for 2 h in liquid MS medium with 100 μM H_2_O_2_ and then treated similarly to the other samples. As a negative control, untreated cells were used.

After incubation, the dye was washed away twice with a fresh MS medium. Then, 10 mg of cells were placed in a 24‐well plate for fluorescent image acquisition using the ZOE Fluorescent Cell Imager microscope (Bio‐Rad). The remaining cells were lysed with a lysis buffer consisting of 2% (w/v) cetyltrimethylammonium bromide (CTAB), 20 mM EDTA, 100 mM Tris‐HCl, pH 8.0, 1.4 M NaCl and 0.5% (v/v) β‐mercaptoethanol (βME). Samples were incubated for 30 min at 65°C and centrifuged at 10 000*g* for 10 min at 4°C. Subsequently, 100 μL of the supernatant were transferred to a 96‐well plate, and the DCF fluorescence signal (excitation/emission 485 nm/535 nm) was measured in endpoint mode using a PerkinElmer EnSpire multimode plate reader.

### 2.6 Cross‐Tolerance Experiments

To assess cross‐tolerance, the different cell cultures were subjected to hypoxia for 48 h, as well as to heat and salt stress for 24 h. Three independent biological experiments were conducted in technical duplicate, for non‐adapted cell populations or adapted cells (10% and 20% PEG). To simulate low‐oxygen environment, cell populations (control, 10% PEG, and 20% PEG adapted cells) were placed in a static incubator at 28°C for 48 h, 4 days after subculturing. An oxygen meter was used to estimate the dissolved oxygen (dO_2_) levels in the medium at 23°C, resulting in 9.33 ± 0.15 mg/L under constant agitation and 5.83 ± 0.15 mg/L after 48 h of static incubation, with a dO_2_ reduction of 37.5%. For heat shock experiments, 4 days after sub‐culturing into fresh medium, the different cell populations were transferred to a rotary shaker in the dark conditions at 37°C for 24 h. Salt stress was induced by adding sodium chloride (NaCl) was added to the culture medium at a final concentration of 100 mM, 4 days after subculturing. Experimental controls were maintained in liquid MS medium on a rotary shaker at 28°C in the dark, for 24 h (salt and heat stress) or 48 h (oxygen limitation).

Cellular viability was determined by fluorescent microscopy (Zeiss Axioplan 2) after a fluorescein diacetate staining. Briefly, 25 mg of fluorescein diacetate was resuspended in 5 mL of acetone and mixed. Then, 10 μL of fluorescein solution was added to 1 mL of cells, and the mixture was incubated in the dark for 10 min. After centrifugation for 3 min at 500*g* at room temperature, the supernatant was removed. The cell pellets were washed with MS (pH 5.8), and resuspended in MS. Cells were then centrifugated for 3 min at 500*g* at room temperature and the pellet was resuspended in glycerol/MS (50%). An aliquot of 200 μL was placed on a slide for microscopy analysis.

After counting the total number of cells and the vital ones in three fields of view, the cellular viability percentage was calculated as it follows:

$$∆V=\frac{\mathrm{VT}-\mathrm{VNT}}{\mathrm{VNT}}\times 100,$$

where,∆V is the difference in viability; VT is the cellular viability percentage in treated cells; VNT is the cellular viability percentage in control cells. Three independent experiments were carried out. Three hundred cells were counted for each experiment.

### 2.7 Plant Growth and Stress Treatments

To investigate *in planta* the gene expression patterns of selected DEPs, young potato shoots grown in vitro (solid MS medium with 3% sucrose) were transplanted in polystyrene trays (5 cm alveolus diameter) containing wet commercial peat (COMPO BIO article number 1152014005) and kept in controlled growth conditions for acclimation (100% humidity, 16‐h light/8‐h dark photoperiod, 24°C–20°C). After 2 weeks, the plantlets were transplanted in bigger pots (15 cm diameter, 13 cm height). After 1 week of acclimation, the plants were exposed to different stresses. Specifically, plants (six for each treatment) were used as control and irrigated with tap water or irrigated with a 250 mM NaCl solution or were deprived of water. Leaf samples from the plants exposed to salt stress and their corresponding controls were collected 7 days post‐stress exposure, monitoring the onset of visible leaf chlorosis and stunted growth. For plants experiencing drought stress and their respective controls, leaf samples were collected after 12 days from water withholding, at which point leaf wilting and a significant decrease in relative water content were observed. Relative water content was calculated on 6 leaves per condition as follows:

$$\mathrm{RWC}(\%)=\frac{\text{FW}-\text{DW}}{\text{TW}-\text{DW}}\times 100,$$

where FW is the fresh weight, DW is the dry weight and TW is the rehydrated weight. All the collected leaf material was immediately frozen in liquid nitrogen for further expression analysis. For each treatment, three independent experiments were carried out.

### 2.8 Expression Analysis of Differentially Expressed Genes Encoding Selected Deps

Total RNA was extracted from the control and treated plants using 1 mL of TRIzol (Thermo Fisher Scientific, Wilmington, DE, United States), according to the manufacturer's protocol. RNA quality and concentration were estimated by agarose gel electrophoresis and NanoDrop ND‐1000 spectrophotometer (Thermo Fisher Scientific, Wilmington, DE, United States), respectively. The first strand cDNA synthesis was carried out using SuperScript III Reverse Transcriptase (Life Technologies, Carlsbad, CA, United States) and random hexamers, starting from 1 μg of DNase‐treated total RNA. Quantitative reverse transcription polymerase chain reaction (qRT‐PCR) was performed in QuantStudio™ 5 Real‐Time PCR Instrument (Thermo Fisher Scientific Inc.). Each PCR reaction consisted of 1 μL of 1: 20 diluted cDNA, 5 μL of 2X PowerUp™ SYBR™ Green Master Mix (Applied Biosystems, CA, United States), and 0.4 μM of each gene‐specific primer in a total volume of 10 µL. Gene‐specific primers' pairs are listed in Table S1. To normalize plant gene expression values, *actin* was used as an internal control. The thermal cycling conditions were 50°C for 2 min (to activate Uracil‐DNA Glycosilase and prevent amplification of carryover DNA fragments), one cycle at 95°C for 2 min, followed by 40 cycles of three steps at 95°C for 15 s, 56°C for 15 s and 72°C for 30 s, as reported in Cappetta et al. (2023). Quantification of gene expression was carried out by the $2^{-\Delta \Delta C_{T}}$ method (Pfaffl 2001) and the relative expression of each gene was expressed by using untreated control as a calibrator. qRT‐PCR reactions were performed for three biological samples and three technical replicates.

### 2.9 Statistical Analyses

Proteomics analysis was performed in three biological replicates for each cell population and, for each quantified protein, the Proteome Discoverer program calculated the Abundance Ratio in each stress condition (as the average of the three biological replicates) over the control sample, together with Abundance Ratio Adj. *p*‐value. The Proteome Discoverer program performed the principal component analysis (PCA) on grouped samples using the Scores Plot for visualization. Statistical comparisons in RT‐qPCR, ROS and metabolite measurements analyses were performed using one‐way ANOVA with GraphPad Prism 8.0 software (San Diego, CA, USA).

### 2.10 Data availability

The mass spectrometry proteomics data were deposited on the public repository ProteomeXchange Consortium via the PRIDE partner (Perez‐Riverol et al. 2022) with the dataset identifier PXD050665.

## 3 Results and Discussion

### 3.1 Potato Proteome Changes in Response to Abrupt and Stepwise Water Deficit Conditions

**Figure 1:**
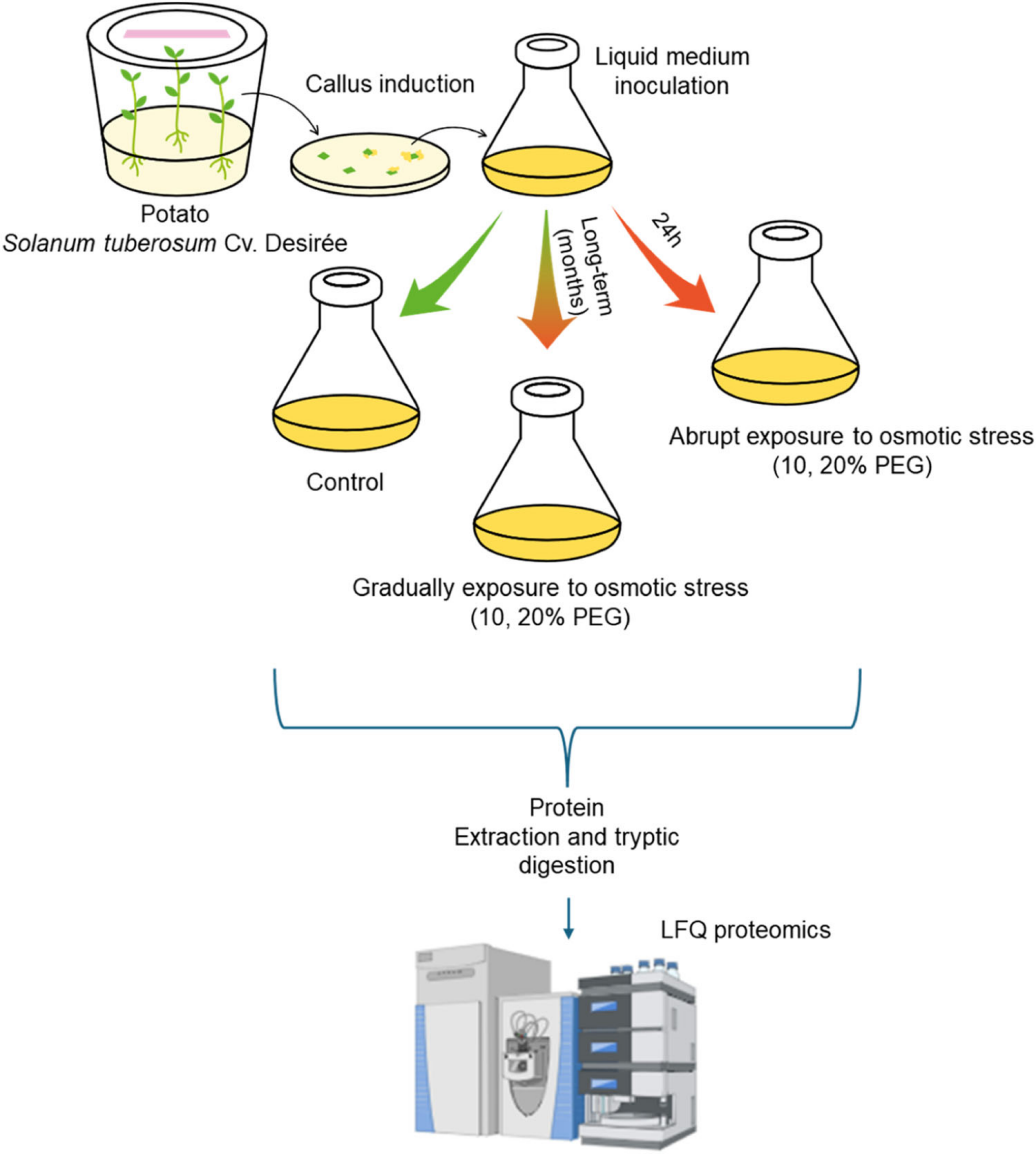
Experimental setup for simulating sudden and gradual exposure to osmotic stress conditions in potato cell cultures. Protein samples upon the different treatments were subjected to Proteomics identification and label‐free quantification (LFQ).

We utilized a slightly modified protocol of our previous in vitro cell model, initially described by Leone et al. (1994a) to simulate both acute and stepwise exposure to PEG‐mediated water deficit in potato suspension cells. Briefly, we prepared potato cell cultures from leaf callus of the Desirée cultivar and established six cell populations, namely two control groups under optimal conditions (one for shock and one for adaptation), two groups exposed to sudden moderate and severe hyperosmotic stress using respectively 10% or 20% (w/v) PEG for 24 h, and two groups gradually adapted to long‐term moderate and severe water stress by incrementally adding PEG until reaching 10% and 20% PEG, respectively (Figure 1). As previously reported (Leone et al. 1994a), adapted cells exhibited cell viability and morphology similar to control cells. Conversely, abrupt osmotic exposure decreased cell viability and modified cell morphology (Figure S1).

To gain a broad understanding of the molecular mechanisms underpinning potato cell responses to water deficit conditions, we carried out a label‐free quantitative (LFQ) mass spectrometry‐based proteomic analysis. Proteins from control, 10% and 20% (w/v) PEG abrupt exposure and 10% and 20% PEG gradual exposure were roughly purified, digested by trypsin, and run in parallel on a nano‐UPLC‐MSMS system. Then, a comprehensive bioinformatic analysis was conducted to compare the statistically significant changes in peptide signal intensity of the identified proteins following the treatments. The total protein groups identified were around 3917 as reported in the Dataset S1 and the DEPs were detailed in the Dataset S2.

Proteomic analysis revealed substantial variations in protein expression profiles among the different potato cell populations. All the statistically significant DEPs are listed in the Dataset S2. The box plots of ungrouped and grouped protein abundance distributions (Figure S2a,b) show consistent medians and similar interquartile ranges across various experimental conditions. Furthermore, PCA (Figure S3) reveals a clear distinction between PEG‐shock and PEG‐adapted conditions, with the separation between moderate and severe scenarios becoming more accentuated under PEG‐shock conditions.

**Figure 2:**
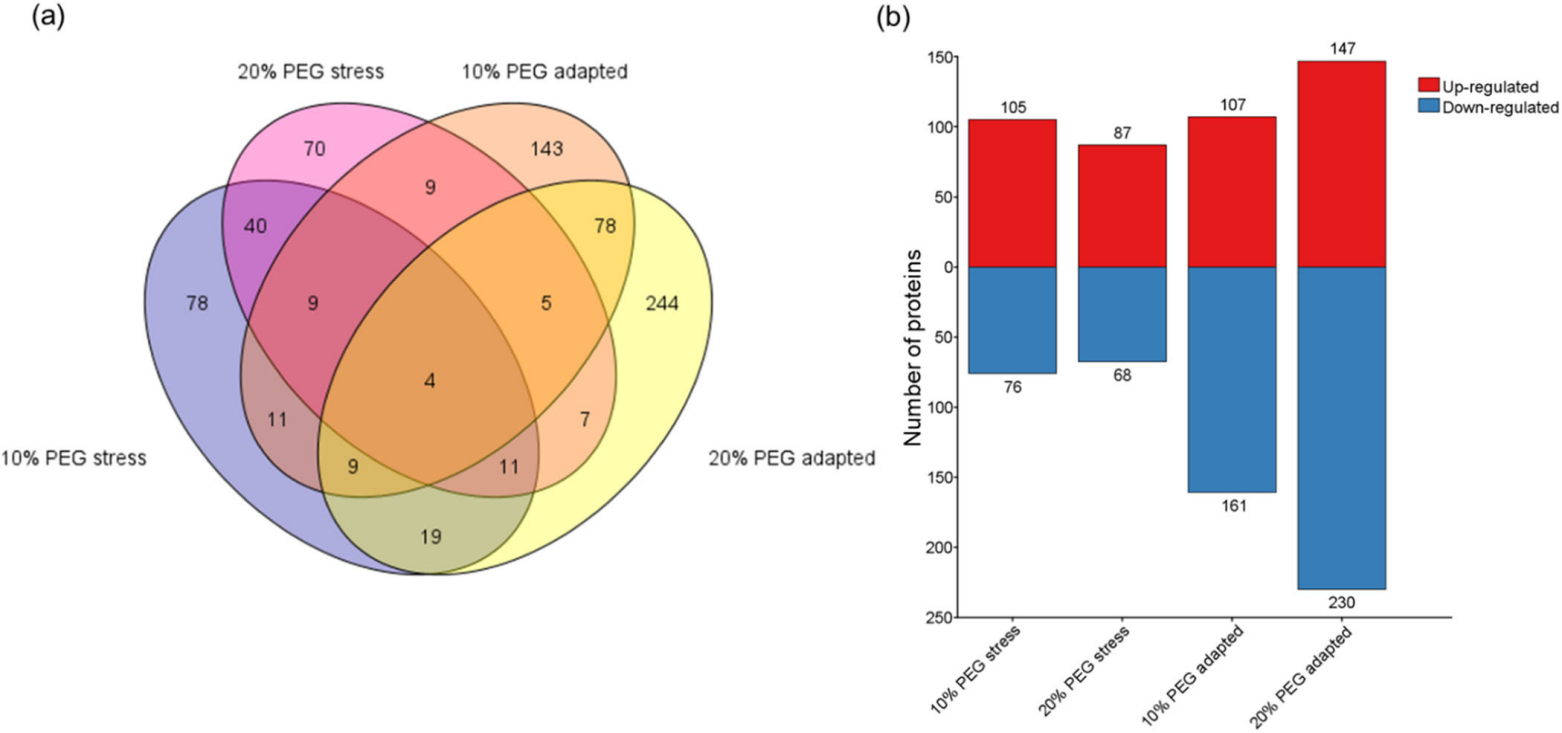
Proteomic data analysis. (a) The Venn diagram illustrates the distribution of identified proteins across four experimental conditions, delineating both the shared and unique proteins. (b) Number of proteins that were upregulated (red) versus downregulated (blue) in each condition. [Color figure can be viewed at wileyonlinelibrary.com]

The Venn diagram (Figure 2a) highlights the divergence in DEPs across conditions, with a marked increase in the number of DEPs observed in cells stepwise exposed to osmotic stress. Specifically, adaptation to 10% PEG was characterized by 143 unique DEPs, whereas adaptation to 20% PEG was marked by 244 unique DEPs. Moreover, a subset of 96 DEPs (78 of which were unique) was found to be common to cells adapted to both 10% and 20% PEG concentrations, suggesting a shared proteomic adjustment mechanism in response to varying degrees of osmotic stress (Dataset S3). Interestingly, among the 96 common DEPS, 43 exhibited upregulation (in both conditions), encompassing proteins pivotal to critical cellular processes such as translation elongation (e.g., Eukaryotic translation initiation factor 5 A, RING‐type E3 ubiquitin transferase, Elongation factor G, acidic ribosomal protein P1a), organelle fusion (e.g., 2 syntaxins) and protein refolding (e.g., 2 heat shock protein 70 and 1 heat shock protein 40). Conversely, in response to abrupt exposures to 10% and 20% PEG, 78 and 70 unique DEPs were identified, respectively, with 64 shared proteins. Among these, 49 were upregulated and mainly associated with processes related to amino acid metabolism and degradation (e.g., aldehyde dehydrogenase, dihydrolipoyl dehydrogenase, 3 phenylalanine ammonia lyase), biosynthesis of secondary metabolites and carbon metabolism (e.g., oxoglutarate dehydrogenase, malate dehydrogenase), suggesting divergent response mechanisms compared to that observed in adapted cells.

Only four DEPs (including two germin‐like proteins) were common across all shocked and adapted cells, indicating a very contracted core set of stress‐response proteins involved both in short‐ and long‐term response to PEG‐induced water stress (Dataset S3).

The number of upregulated versus downregulated proteins within the shocked and adapted cells are displayed in Figure 2b. At 10% PEG shock, 105 proteins were upregulated and 76 were downregulated; at 20% PEG shock, the numbers decreased to 87 upregulated and 68 downregulated proteins. In the adapted cells, at 10% PEG, there were 107 upregulated and 161 downregulated proteins, while at 20% PEG, a substantial increase to 147 upregulated and 230 downregulated proteins was observed. These data suggest that a broader proteome reprogramming is necessary to mitigate the effects of prolonged and increasing osmotic stress. Moreover, these findings show that potato cells employ a complex array of molecular strategies for immediate or long‐term responses to osmotic stress, with distinct patterns of protein regulation emerging between shock and adaptation phases.

### 3.2 GO Enrichment Analysis Unveils Differential Response Mechanisms of Potato Cells to Gradual and Abrupt Water Deficit

**Figure 3:**
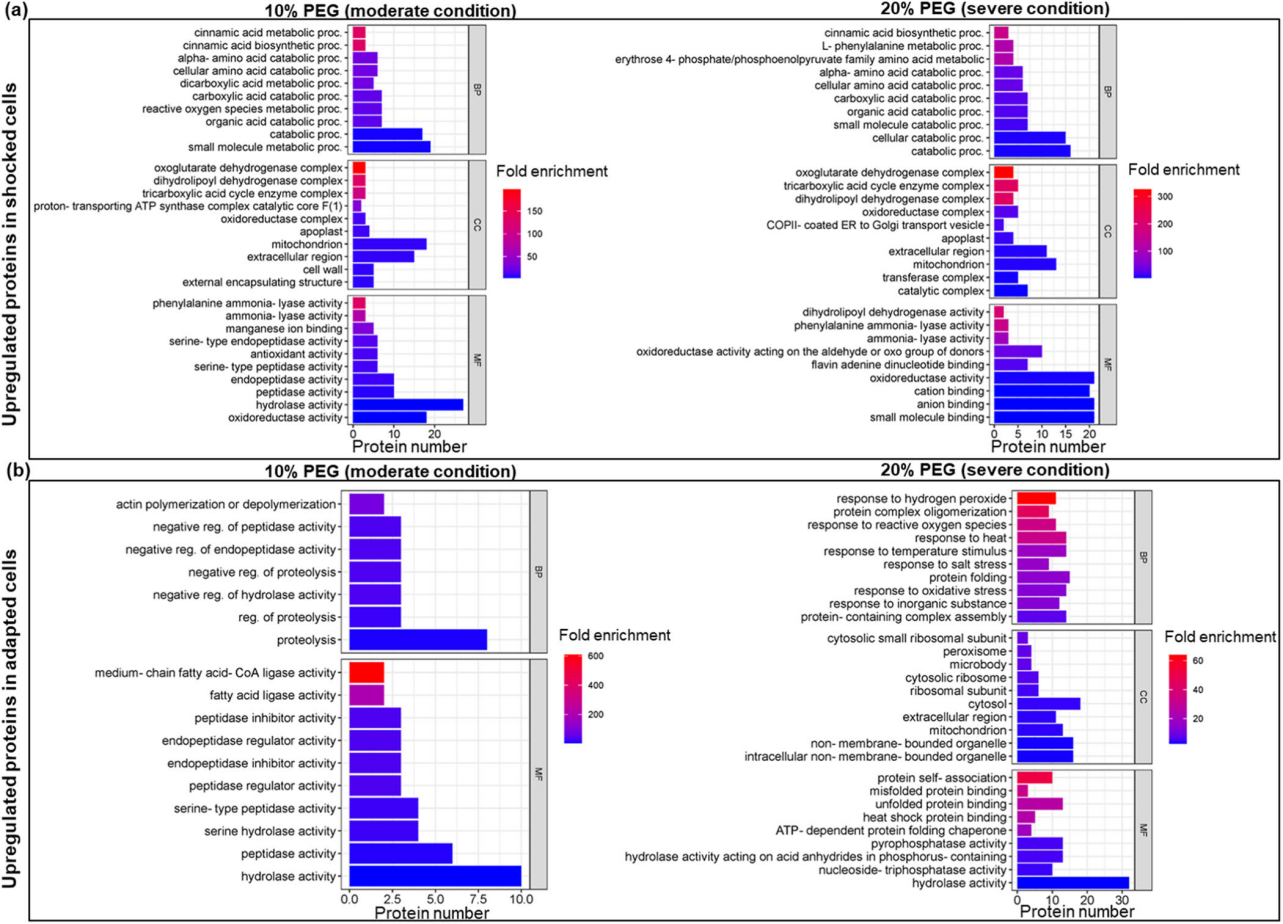
Gene ontology (GO) enrichment analysis of upregulated proteins in cells under shock (a) and gradual adaptation (b) to water deficit conditions. [Color figure can be viewed at wileyonlinelibrary.com]

**Figure 4:**
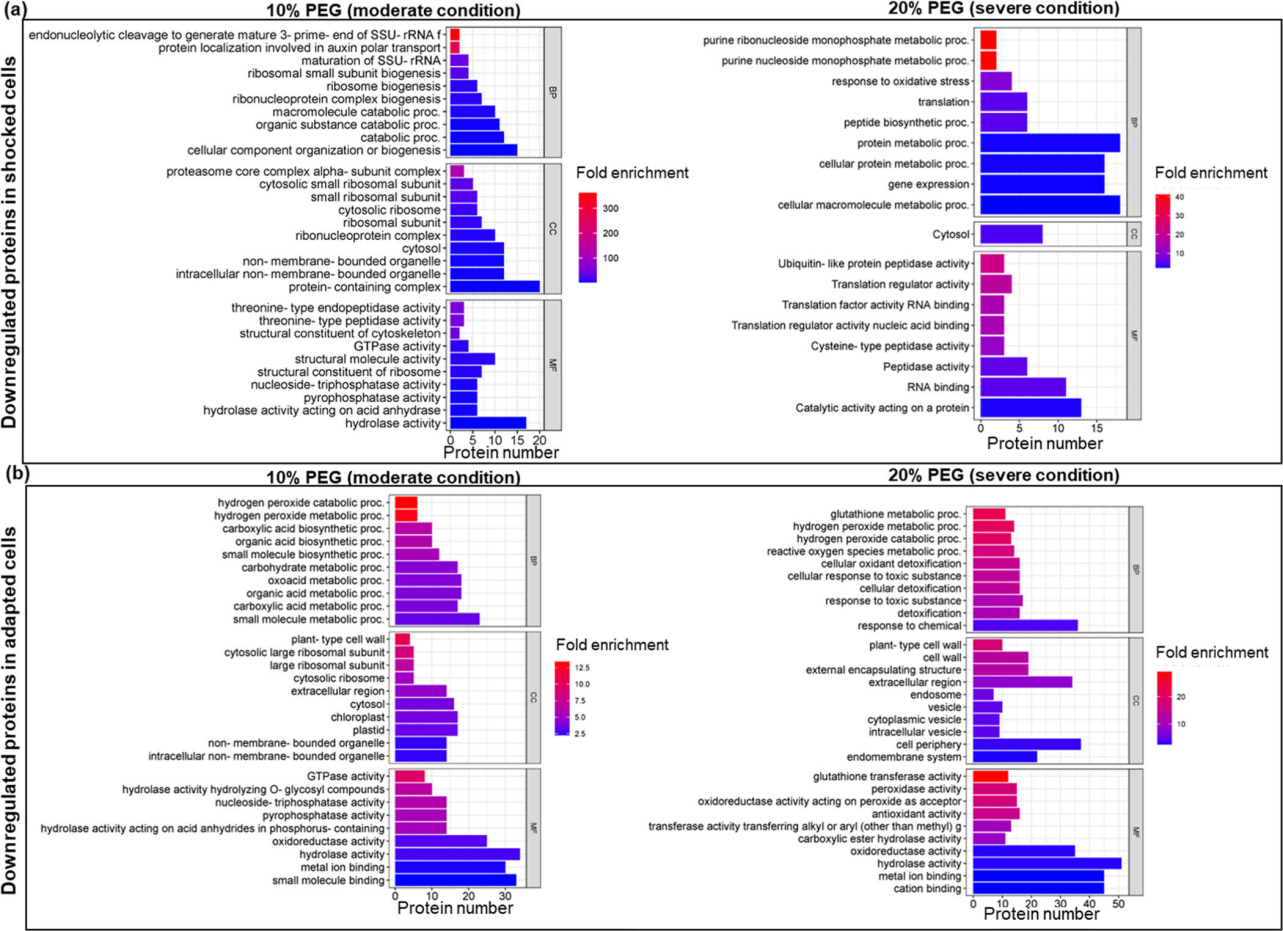
Gene ontology (GO) enrichment analysis of downregulated proteins in shock (a) or adapted cells (b). [Color figure can be viewed at wileyonlinelibrary.com]

To investigate the molecular functions of DEPs under varying intensities and durations of osmotic stress, we performed an enrichment analysis of GO terms (Dataset S4). The results are displayed in Figure 3, which illustrates enriched GO terms of upregulated proteins, and Figure 4, which focuses on GO of proteins downregulated in the different experimental conditions.

In response to acute moderate and severe water stress, potato cells exhibit a rapid upregulation of proteins associated with the metabolism and biosynthesis of cinnamic acid such as caffeoyl‐CoA O‐methyltransferase 4‐coumarate‐‐CoA ligase 2, phenylalanine ammonia‐lyase (PAL), tyramine hydroxycinnamoyl transferase and caffeoyl‐CoA O‐methyltransferase. The combined activity of these enzymes likely leads to an altered balance of metabolites within the phenylpropanoid pathway, including flavonoids and lignins, that are known to play critical roles in plant growth, development and defense (Khawula et al. 2024; Liu et al. 2022; Nicolas‐Espinosa et al. 2023).

Alpha‐amino acid catabolic processes and cellular amino acid catabolic processes are enriched in moderate and severe abrupt stress, indicating altered metabolic activity during the stress response. Key proteins such as aspartate aminotransferase, glutamate dehydrogenase, oxoglutarate dehydrogenase and dihydrolipoyl dehydrogenase are involved in linking amino acid metabolism with energy production pathways. The potential metabolic shift is further reflected in the enrichment of specific cellular component GO categories, such as the oxoglutarate dehydrogenase complex, TCA cycle enzyme complex and dihydrolipoyl dehydrogenase complex, which are essential for converting amino acid‐derived intermediates into ATP during periods of increased metabolic demand. These enzymes and complexes are involved in various processes, such as energy production, metabolism and detoxification of ROS as widely reported (Igamberdiev and Bykova 2023; Kapoor et al. 2015).

Moreover, the enrichment of peptidase activity GO terms including subtilisin‐like proteases and aspartic proteinases suggests a significant activation of protein turnover under moderate stress conditions (10% PEG stress).

Gradual adaptation to osmotic stress reveals a distinct profile of upregulated proteins compared with the immediate stress response, especially under severe conditions (20% PEG). Under moderate conditions, a predominant enrichment of terms related to peptidase activities was observed. However, proteins upregulated during severe conditions showed a sustained activation of responses to various stressors (Figure 3b). The activation of key proteins such as ascorbate peroxidase (M1B3Q2), glutathione transferase (M0ZNL8), catalase (M1ALT0), ATP synthase subunit alpha (M1C0X2), gamma‐aminobutyrate transaminase (M1CD67), likely contribute to mitigate the oxidative stress. Furthermore, the involvement of heat shock proteins and chaperonines (M1CYA5, M1DJ69, M0ZU24) enhances the plant cell ability to cope with long‐term water deficit by preserving protein folding (Hartl, Bracher, and Hayer‐Hartl 2011; Landi et al. 2019).

On the other hand, among the downregulated processes in shocked cells (Figure 4a), there was a marked repression of proteins involved in endonucleolytic cleavage to generate mature 3'‐end of SSU‐rRNA, and ribosome and ribonucleoprotein complex biogenesis. Interestingly, the processes involved in transcriptional and translational activity were repressed. These results are consistent with our previous findings, revealing extensive repression of transcripts encoding various ribosomal proteins and impacting processes related to transcription and protein synthesis in potato cells subjected to acute water stress (Ambrosone et al. 2017). Consistently, cellular component and molecular function categories related to GTPase activity, peptidase activity and structural molecule activity, particularly structural constituents of the ribosome, also show decreased representation in shocked cells under both moderate and severe conditions. The repression of structural molecule activity under different abiotic stress conditions has been previously reported for several plant species (Merchante, Stepanova, and Alonso 2017).

Gradual adaptation is characterized by downregulation of a subset of proteins belonging to detoxification, hydrogen peroxide catabolic process, glutathione metabolism, peroxidase and antioxidant activity categories, which are particularly pronounced 20% PEG adapted cells. The proteins involved in these processes include several peroxidases (M1C3C2, M1AP83, M1A5B5, M1C911, M0ZIL5, M1B986, M1CE55, M1B8Q1), glutathione transferases (M0ZSQ8, M0ZRT5, M0ZQ26, M0ZQ38, M1BWS6, M1D2J3, M1B647, M1ANN8), and catalase isozyme (P55312).

Collectively, GO analyses delineate a clear distinction between the immediate acute stress response and the long‐term adaptation mechanisms of plant cells to osmotic stress. During the initial shock phase, cells rapidly upregulate processes associated with primary and secondary metabolism and strongly repress translation‐related processes. These responses likely aim at rapidly mitigating the detrimental impacts of high osmotic pressure, water deficit and the associated oxidative burst. By contrast, the gradual adaptation of potato cells reveals a strategic shift toward enhancing and permanently activating responses to various abiotic stresses, alongside the upregulations of protein categories involved in protein folding. This shift may represent a sophisticated adaptation strategy of cells for maintaining protein functionality and structural integrity over time.

### 3.3 KEGG and MapMan Analyses Disclose Shared and Unique Molecular Pathways in Shocked Versus Adapted Cells

To dissect the common and divergent pathways activated in shocked and adapted cells, we conducted an in‐depth investigation of the Kyoto Encyclopedia of Genes and Genomes (KEGG)‐enriched pathways (Table S2).

**Figure 5:**
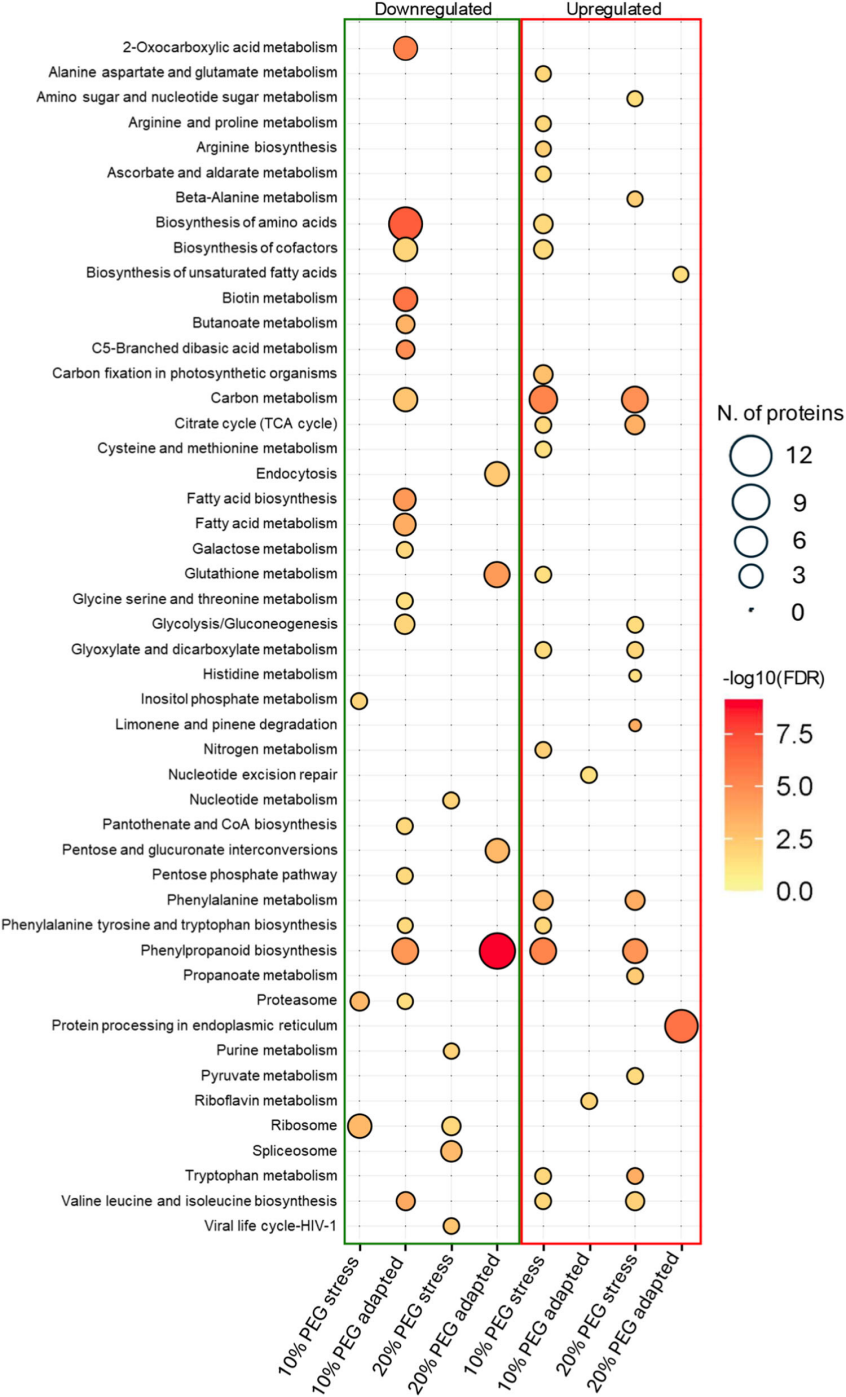
The KEGG pathway enrichment analysis. Y‐axis shows the overrepresented KEGG‐enriched pathways in shocked and adapted cells. The dot size and color represent the protein number and −log10 FDR value, respectively. The low FDR values are in yellow, and the high values are in red. [Color figure can be viewed at wileyonlinelibrary.com]

Upon abrupt exposure to 10% water‐limiting conditions, the KEGG pathway analysis revealed a substantial upregulation in pathways that may be involved in plant's stress response and energy management, as illustrated in Figure 5. In particular, the phenylpropanoid biosynthesis pathway was particularly enriched due to the upregulation of six proteins (four peroxidases and two phenylalanine ammonia‐lyses) (Table S3). Consistently, the phenylalanine pathway was also found to be activated in shock conditions. Interestingly, many proteins belonging to these pathways were conversely repressed during the gradual adaptation. Specifically, we observed the downregulation of 11 proteins in 20% adapted conditions, including nine peroxidases, one PAL and one catechol‐O‐methyltransferase, as detailed in Table S3 and Figure 4. These findings substantiate GO enrichment results and are coherent with previous studies indicating that plants, in response to abiotic stresses (such as salt, drought or oxidative), redirect the metabolic flux (i.e., phenylpropanoids), likely to attenuate cellular damages (Dixon and Paiva 1995; Francini, Giro, and Ferrante 2019; Oliva et al. 2021; Pandey, Ramegowda, and Senthil‐Kumar 2015; Sharma et al. 2022).

An increase in metabolic pathways related to specific amino acids such as valine, leucine and isoleucine, as well as in pathways related to alanine, aspartate and glutamate, tryptophan, arginine and proline metabolism, was also observed in shocked cells. These findings indicate substantial changes in amino acid profile, which has been widely reported in plants exposed to osmotic stress (Kumar et al. 2021; Oliva et al. 2021; Fouad et al. 2023).

Additionally, the carbon metabolism pathway is also activated, reflecting the need for rapid energy reallocation when cells face abrupt stress, in both moderate and severe conditions (Figure 5 and Table S3). These data corroborate insights derived from our GO analysis highlighting the central role of this pathway in the abrupt response to water stress.

Concurrently, the KEGG pathway analysis revealed a downregulation of ribosome pathway, coherent with GO analysis together with a repression of critical posttranscriptional and posttranslational processes such as the proteasome and spliceosome during mild and severe shock osmotic conditions (Figure S5).

Regarding the adaptive response, our data confirm that potato cells deploy distinct strategies depending on the severity of the stress as evidenced by our GO analysis. The 10% adapted condition showed an upregulation of the riboflavin metabolism pathway, suggesting that this molecule may play an important function in helping plants adapt to stress. Riboflavin is known to contribute to various critical functions, including energy metabolism, antioxidant activity and acting as a signaling molecule in plant defense mechanisms (Nie and Xu 2016; Tian et al. 2022). Future works will aim to quantify the role of this vitamin in our experimental conditions and determine its potential role in potato stress adaptation.

Conversely, key metabolic processes such as amino acid biosynthesis and carbon metabolism, which were significantly upregulated under shock conditions, were notably downregulated, suggesting that gradual adaptation allows cells to achieve a new homeostasis and restore normal cellular functions, as previously reported (Osakabe et al. 2014; Bielach, Hrtyan, and Tognetti 2017; Seleiman et al. 2021).

As for adapted cells, a notable downregulation was also observed in the pentose and glucuronate interconversions and endocytosis pathways. Specifically, three pectinesterase and two pectate lyases belonging to the pentose and glucuronate interconversions pathway were downregulated; an ethylene‐responsive protein, a stromal membrane‐associated protein, a developmental protein, the vacuolar protein35 and RabA2 belonging to the endocytosis pathway were downregulated. Repression of these processes suggests alterations in carbohydrate metabolism and cellular transport processes upon stress adaptation. Mirroring the patterns identified in the GO results, this downregulation may indicate a reshuffling of metabolic activities to better cope with a more stringent water stress.

Interestingly, there was a pronounced upregulation in the protein processing in the endoplasmic reticulum pathway, including 9 heat shock proteins (HSPs) belonging to the small heat shock protein (HSP20) family, corroborating the GO outcomes, and confirming the key role of proteins preventing cellular structural damages during the adaptation phase.

Worth mentioning our KEGG analysis also reveals a significant alteration in fatty acid biosynthesis and metabolism in adapted cells. Specifically, we observed an activation of the biosynthesis of unsaturated fatty acids (UFAs) with the upregulation of an Acyl‐CoA thioesterase and the 3‐ketoacyl CoA thiolase 2 in the 20% adapted. This regulation might also provide mechanistic information regarding the production of UFAs observed in adapted cells, which have been reported to modulate membrane fluidity as part of their adaptation strategy under water deficit, as previously described (Leone et al. 1996).

**Figure 6:**
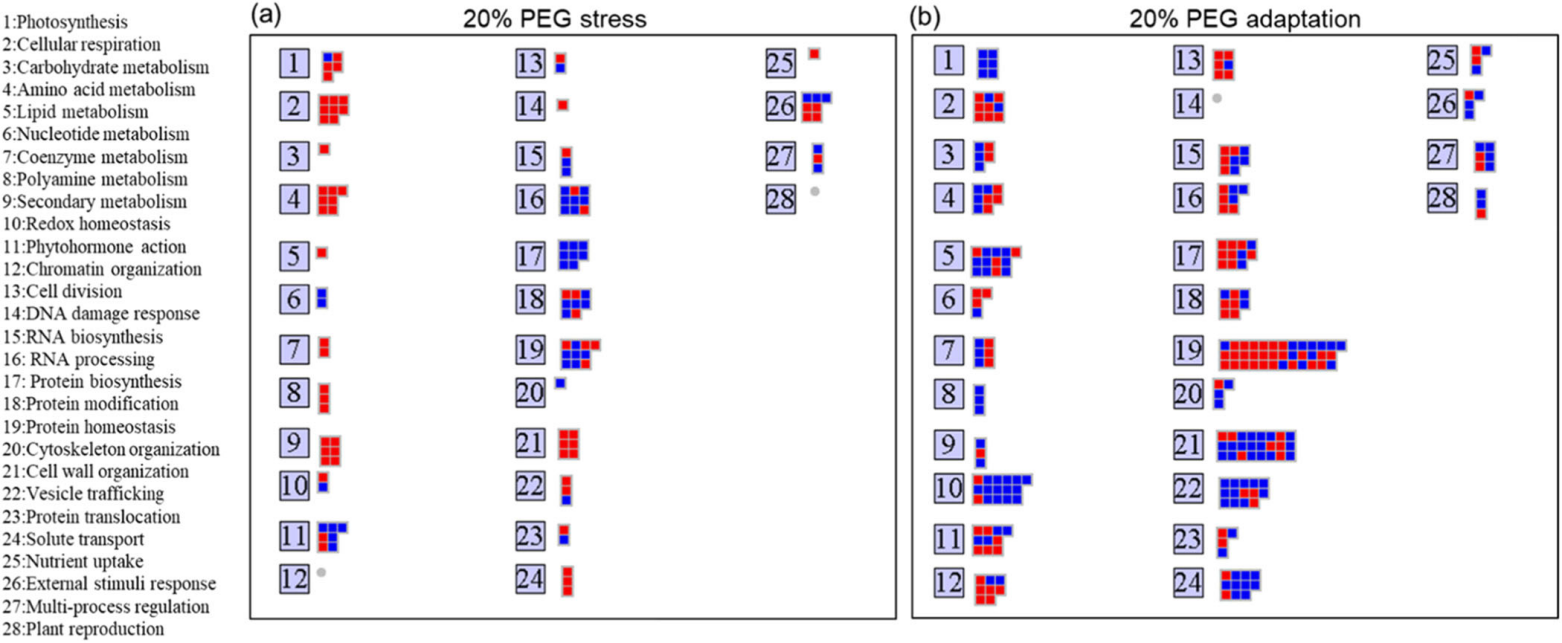
MapMan pathway (bin) representation comparing up‐ (red) and down‐ (blue) regulated proteins in PEG 20% shocked and adapted cells. [Color figure can be viewed at wileyonlinelibrary.com]

The MapMan analysis carried out on the protein datasets of 20% PEG‐shocked and adapted cells corroborated and extended our knowledge of the cellular processes supporting the enhanced resilience of potato cells to osmotic stress. Specifically, the analysis identified an enrichment and activation of pathways involved in RNA and protein processing, biosynthesis, homeostasis, chromatin organization and cell division within cells adapted to 20% PEG conditions (Figure 6).

DEPs included in these selected pathways are included in the Dataset S5. More in detail, the protein homeostasis is the most abundant category with 24 upregulated and 12 downregulated proteins. To further understand the regulatory processes governed by the identified 24 upregulated proteins we constructed a protein–protein interaction (PPI) network using the STRING database (https://string-db.org/), based on the integration of various approaches, including text mining, databases, experimental validation, co‐expression and neighborhood interactions. Intriguingly, within the upregulated proteins we identified a cluster of eight proteins with chaperone functions (Figure S6). This cluster exhibited strong relationships and enriched functional categories associated with the protein folding, cellular component organization as well as response to abiotic stress (e.g. heat, salt) (Figure S6b).

MapMan results also showed that several DEPs mapped in the cell division category are upregulated in adapted cells (e.g., the replication factor RPA2 and the condensin‐II‐specific component CAP‐D2), that likely may sustain cell proliferation under non‐permissive conditions, such as in response to a severe osmotic stress.

Complementing the results from KEGG pathway analysis, the MapMan framework shed light on a remarkable shift in proteins related to chromatin organization, epigenetic regulators and other key components of RNA transcription machinery in 20% PEG‐adapted cells. Remarkably, within the chromatin organization category are several histone deacetylases belonging to subfamilies 1 and 2, enzymes that have been implicated in the epigenetic regulation of drought‐responsive memory genes (Gallusci et al. 2017; Abdulraheem et al. 2024) suggests an adaptive transcriptional response through chromatin remodeling, which enables a sustained reaction to persistent osmotic stress conditions (Kambona et al. 2023). Coherently, we observed in the RNA biosynthesis category (BIN 15) an enhanced expression of NRPD7 and NRPE7, key components of RNA polymerase IV and V complexes, respectively, which are known to play critical roles in the regulation of gene expression through siRNA‐directed DNA methylation and transcriptional gene silencing in plants (Onodera et al. 2005; Rymen et al. 2020). The upregulation of NRPD7 and NRPE7 under long‐term osmotic stress suggests an increased activity in pathways that modify the epigenetic landscape, leading to alterations in gene expression patterns essential for stress adaptation. Notably, in the RNA biosynthesis category, we also found a downregulation of three transcription factors belonging LIM family. Plant LIM proteins are known to interact with actin proteins to regulate the actin cytoskeleton structure (Papuga et al. 2010). However, several studies have reported their significant role in regulating the expression of genes related to the phenylpropanoid biosynthesis pathway (Srivastava and Verma 2017). For example, in tobacco (*Nicotiana tabacum*), the *Nt*LIM1 protein binds to the PAL box in the promoter region to activate the *PAL* gene, a key enzyme in lignin metabolism (Kawaoka and Ebinuma 2001). Further investigation is needed to clarify the specific role of LIM transcription factors in the regulation of the phenylpropanoid biosynthesis pathway within our experimental model.

Collectively, such alterations in transcriptional, posttranscriptional and epigenetic regulation drive global gene expression changes and equip the plant cells with a stable and effective capacity to cope with prolonged osmotic stress conditions.

### 3.4 Functional Validation of Proteomic Data Through the Quantification of Selected Metabolites and ROS Levels

**Figure 7:**
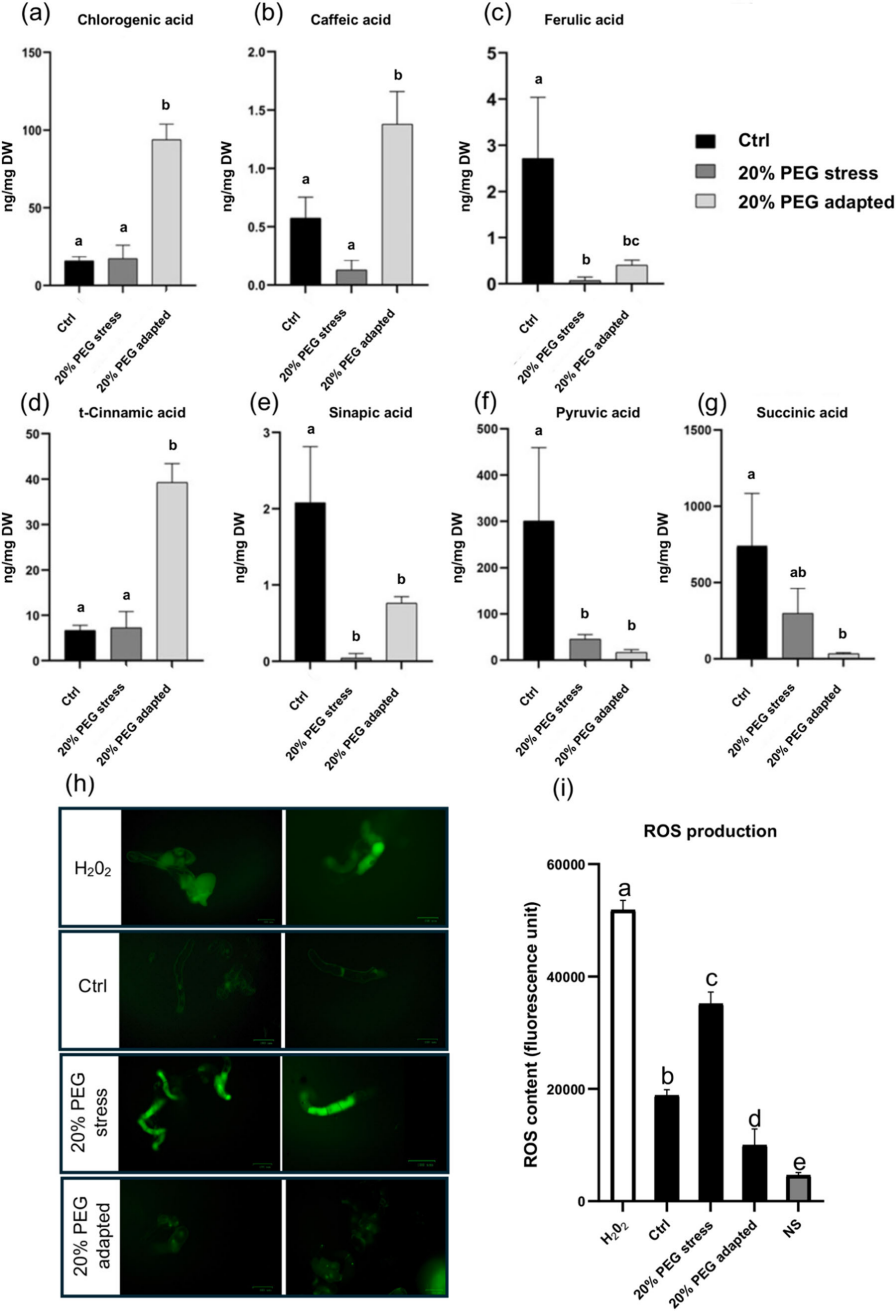
Quantification of selected metabolites and reactive oxygen species levels. (a–g) LC‐ESI‐MS quantification of major phenylpropanoid metabolites (a–e) and organic acids (f,g) in cell samples from control, 20% PEG stress and 20% PEG adapted conditions. (h) Visualization of intracellular ROS using DCFH‐DA staining and the ZOE Fluorescent Cell Imager microscope. The figure includes images of H_2_O_2_‐treated cells, and cells under control, 20% PEG stress and 20% PEG‐adapted conditions. (i) Quantitative measurement of ROS levels via fluorescence intensity in lysed cell extracts, utilizing a PerkinElmer EnSpire multimode plate reader. The values represented in all histograms were obtained from three replicates, and the error bar represents the standard deviation. Different letters on top of histograms indicate statistically significant differences among groups (*p* < 0.05). H_2_O_2_, hydrogen peroxide; NS, Not stained; DW, dry weight. [Color figure can be viewed at wileyonlinelibrary.com]

To functionally validate the proteomic data, we assessed the levels of selected metabolites and ROS, focusing on the most divergent mechanisms observed under stressed and adaptation conditions. As illustrated in Figure 7, the content of cinnamic acids, chlorogenic acids and caffeic acids was significantly higher in adapted cells compared with control and shocked potato cells. It is plausible to hypothesize that the observed repression of enzymes (e.g., Catechol O‐methyltransferase and some peroxidases) involved in phenylpropanoid pathways in adapted cells could lead to the accumulation of these phenolic acids. Specifically, Guo and collaborators (2001) demonstrated that the downregulation of caffeoyl CoA 3‐O‐methyltransferase (CCOMT) results in the accumulation of soluble caffeic acid β‐d‐glucoside in transgenic alfalfa plants (Guo et al. 2001). Similar findings were observed in transgenics alfalfa and *Arabidopsis*, where downregulation of hydroxycinnamoyl CoA:shikimate/quinate hydroxycinnamoyltransferase (HCT), the enzyme that catalyzes the conversion of p‐coumaroyl‐CoA to p‐coumaroyl shikimate, lead to an accumulation of flavonoids (Hoffmann et al. 2004; Shadle et al. 2007). Additionally, the accumulation of cinnamic acid and its derivatives is known to negatively affect the synthesis and activity of PAL in potato and other crops (Lamb and Rubery 1976; Mavandad et al. 1990), in line with our protein expression data.

The difference between the rapid activation of the phenylpropanoid pathway during acute stress and the delayed accumulation of metabolites like cinnamic acid during adaptation highlights complex metabolic regulation. Acute stress may increase turnover or degradation, hindering immediate accumulation. Additionally, repression of key enzymes during adaptation slows metabolite conversion of cinnamic acid and other phenolic acids, allowing their accumulation. Further studies, including time‐course analyses, are needed to elucidate the regulatory dynamics that govern the relationship between enzyme activity and metabolite levels in potato cells.

Phenolic acids are known to mitigate abiotic stress responses by influencing various processes, such as ROS scavenging, hormonal crosstalk and the regulation of stress‐related genes (Bistgani et al. 2019; Chen et al. 2019; Šamec et al. 2021). According to these findings, our data suggest that phenolic acids may provide advantages for long‐term response to osmotic stresses. Metabolic engineering approaches are needed to fully understand the regulation of this metabolic flux under potato cell adaptation.

Then, we quantified the levels of pyruvic acid and succinic acid as the metabolic energy pathways appeared to be differentially regulated in stressed and adapted cells. Results reported in Figure 7f,g show that the level of these organic acids are generally low under non‐optimal growth conditions. However, we observed a higher content of pyruvic acid (2.7‐fold increase) and succinic acid (8.8‐fold increase) in shocked cells than in adapted cells, confirming that adapted cells experience a substantial metabolic shift with respect to stress response, reflecting different energy utilization strategies to cope with abrupt stress or long‐term exposure to water deficit. These elevated levels in shocked cells suggest a more immediate and intense metabolic response, whereas the lower levels in adapted cells may reflect a shift toward more sustained, long‐term metabolic strategies for coping with long‐term exposure to stress.

Finally, to evaluate oxidative stress levels within the potato cell population, we measured the levels of reactive oxygen species (ROS) using the 2',7'‐dichlorofluorescein diacetate (DCFH‐DA) assay. Data presented in Figure 7h–i reveal a clear divergent ROS profile between stressed and adapted cells. Stressed cells exhibit a significant accumulation of ROS, while adapted cells show ROS levels lower than the control group. This aspect is particularly relevant showing that adapted cells adopt effective strategies to control oxidative stress avoiding their long‐term detrimental effects.

In conclusion, these findings not only validate the robustness of our proteomic analysis but also unveil metabolic alterations within the adopted experimental model, paving the way for further functional investigations.

### 3.5 Cross Stress Tolerance in Gradually Adapted Potato Cells to Osmotic Stress

Our comprehensive analysis encompassing GO function annotation, KEGG and MapMan pathway enrichment suggests that long‐term adapted potato cells activate a multifaceted cellular response that may provide a broad‐spectrum resilience, also known as cross‐tolerance.

**Figure 8:**
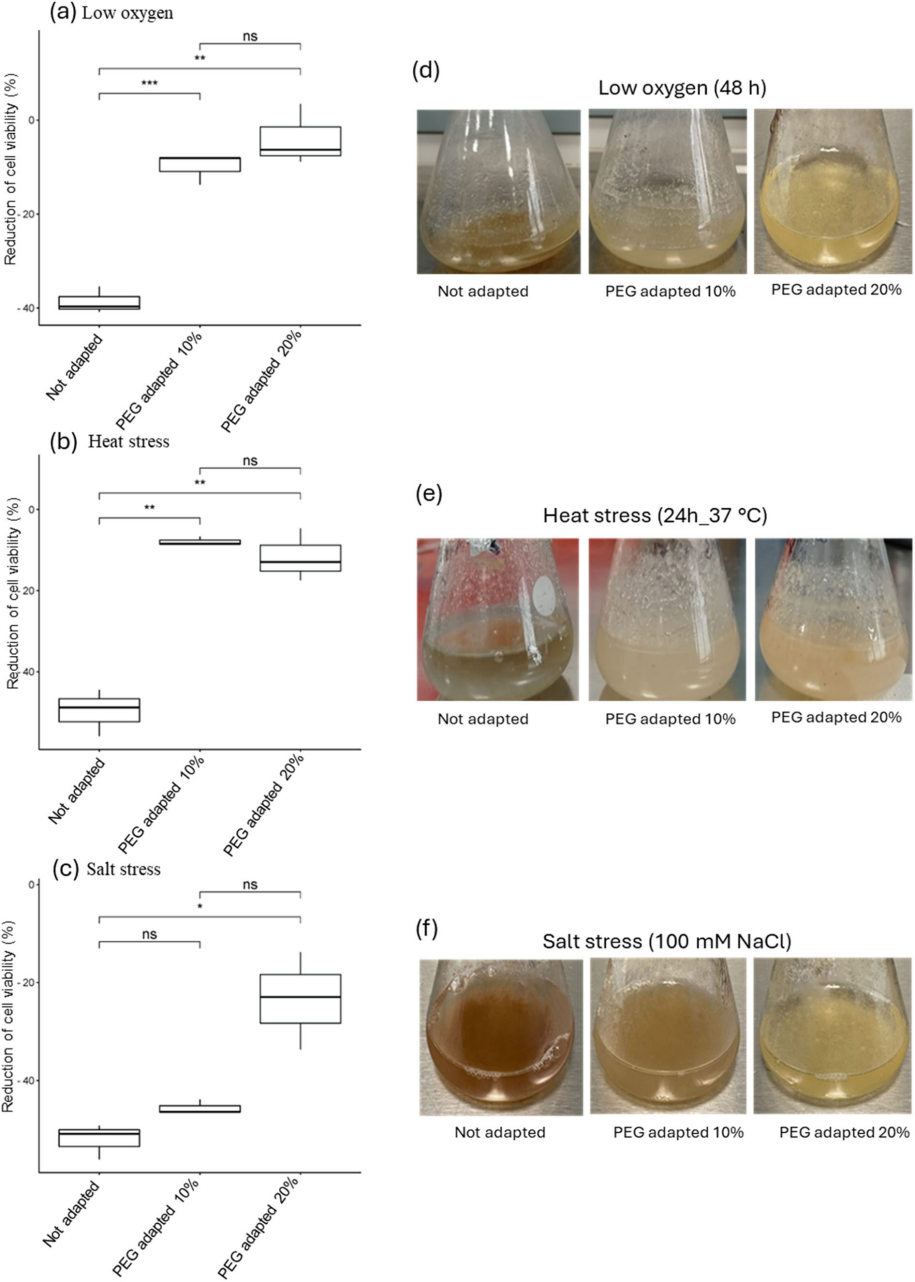
Percentage of reduction of cell viability (a–c) and potato cell cultures (d–f) in control (not adapted) and adapted cells (10% and 20% PEG) after exposure to hypoxia, and heat and salt stress conditions. Asterisks indicate a significant difference among all conditions (*p < 0.05; **p < 0.01; ***p < 0.001 ANOVA test). [Color figure can be viewed at wileyonlinelibrary.com]

To prove this hypothesis, cell viability of non‐adapted (control) and adapted cells (10% and 20% PEG) was estimated upon exposure to oxygen limitation (48 h), heat stress (37°C, 24 h) and salt stress (100 mM NaCl, 24 h) (Figure 8a–c). Interestingly, non‐adapted cells demonstrated a relative reduction in cell viability declining by 39%, 49.5% and 52% under low‐oxygen environment, thermal and salt stress, respectively. In contrast, adapted cells were more tolerant to these stressors. Specifically, the relative reduction in cell viability was 9.8%, 7.9% and 45.6% for the 10% PEG‐adapted cells, and even lower at 3.9%, 11.6% and 23.2% for the 20% PEG‐adapted cells when exposed to oxygen limitation, heat and salt stresses, respectively (Figure 8a–c). The improved response of adapted potato cells to the tested stressors was also highlighted by their healthy aspect compared with non‐adapted cells which, instead, appeared brownish when exposed to the same stress conditions (Figure 8d–f).

The mechanisms underlying this cross‐tolerance are likely multifactorial, involving a network of stress response pathways that are primed by the initial osmotic stress. The adaptation to these stresses may involve the expression of specific proteins that not only protect potato cells against osmotic imbalance but also prepare the cellular machinery to forefront more effectively other types of stressors more effectively as reported for several plant species (Munné‐Bosch and Alegre 2013; Li and Liu 2016; Sharma et al. 2022; Kambona et al. 2023). This could include the upstream activation/repression of genetic and epigenetic regulators, the maintenance of an active antioxidant systems that mitigate oxidative burst and cellular damages, the accumulation of osmolytes as well as chaperone proteins that might be involved in a general stress response to counterbalance misfolded proteins, known to be generated by different types of stress.

### 3.6 From Cells to Plants: Profiling the Gene Expression Patterns of Selected DEPs Under Drought and Salt Stress

DEPs implicated in transcriptional, posttranscriptional and posttranslational processes, along with those involved in metabolic and fatty acid pathways, appear to be key elements of the adaptive mechanism observed in potato cells. To investigate deeper the expression data in potato plants exposed to osmotic stresses, we undertook both regulatory sequence and gene expression analyses on a select group of six genes encoding candidate proteins associated with these key processes. The target genes were selected based on the expression patterns of corresponding proteins that demonstrated unique roles in the mechanisms of adaptation to osmotic stress responses.

More in detail, two heat shock proteins, HSP 17.4 (UniProtKB accession M1AIV3; gene ID: 102591190) and HSP 18.2 (UniProtKB accession M0ZT86, gene ID: 102606179) belonging to the small heat shock protein (HSP20) family and the syntaxin member of the SNARE proteins SYP121 (UniProtKB accession M1CQ32, gene ID: 102587324), already previously identified as an interesting candidate in PEG potato‐adapted cells (Ambrosone et al. 2017), exhibited consistent upregulation in adapted cells and downregulation in shocked cells. This dual response clearly indicates that gradual adaptation to water stress induces proteins involved in maintaining protein stability and cellular homeostasis (Luo, Shi, and Xiang 2022). As discussed above the role of HSPs in abiotic stress is well documented, interestingly the identification of SYP121 as upregulated proteins in adapted potato cells indicated a primary role in the response to gradual adaptation, beyond its well‐known function in vesicle trafficking and involvement in biotic stress response (Hachez et al. 2014).

Additionally, the histone deacetylase HDAC2 (M0ZQ89, gene ID: 102593361) displayed alterations marked by upregulation during adaptation, suggesting their specific roles in the adaptive restructuring of cellular machinery. Also, the 3‐ketoacyl‐CoA thiolase Kat2 (UniProtKB accession M1CWA6, gene ID: 102578706), a key player in fatty acid metabolism, showed exclusive upregulation in potato cells exposed to gradual water stress, corroborating the potential link between fatty acid metabolism and the adaptive response to water‐limited conditions, as suggested by our KEGG and MapMan analyses and our previously reported findings (Leone et al. 1996; Ambrosone et al. 2017). Conversely, the PAL8 (UniProtKB accession M1C5K7, gene ID: 102582618), the pivotal enzyme in phenylpropanoids metabolism, exhibited an opposite behavior with upregulation in shocked cells and downregulation in adapted cells, indicating active production of stress‐related secondary metabolites during abrupt exposure to water‐limited conditions as discussed above. Worth mentioning, 14 *PAL* genes in *Solanum tuberosum* were recently identified using a Genome‐wide analysis, with the *PAL8* specifically involved in the potato response mechanism to high temperature and drought stress (Mo et al. 2022).

**Figure 9:**
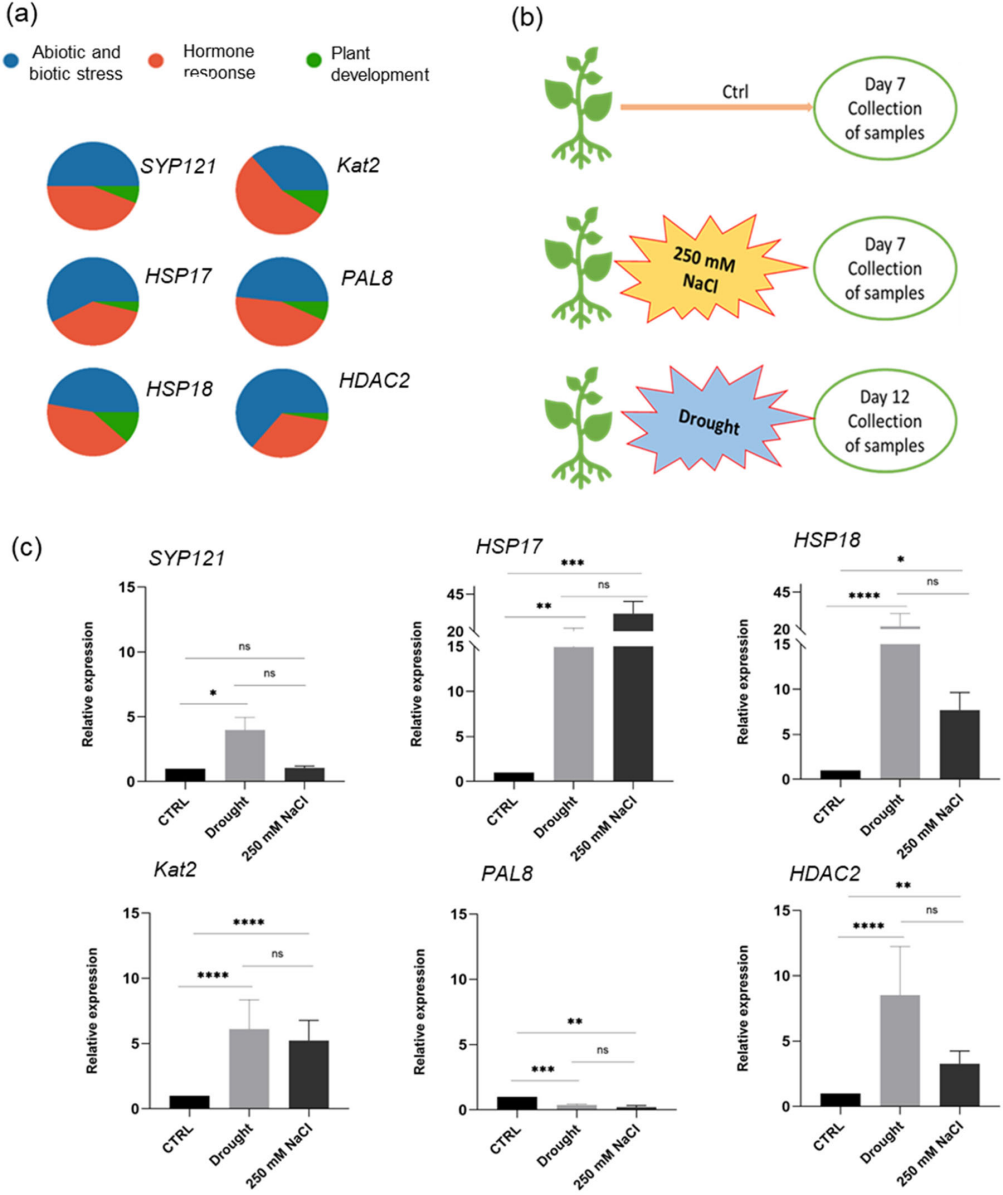
(a) *Cis*‐acting regulatory elements in the promoters of gene encoding selected proteins. Elements were grouped in abiotic/biotic stress responses, hormone‐sensitive and plant development. The total number of *cis*‐acting regulatory elements belonging to each class was expressed as a percentage (%). A Pie matrix showing the % in each promoter was then drawn. (b) Experimental setup for validation of gene expression patterns in potato plants. (c) Expression levels of the six selected genes in leaves of control or stressed potato plants. Columns indicate the mean relative gene expression levels of three replicates ± standard deviation (*n* = 3), determined by RT‐qPCR. Potato actin served as endogenous control. Asterisks indicate a significant difference among all conditions (*p < 0.05; **p < 0.01; ***p < 0.001; ****p < 0.0001 ANOVA test). [Color figure can be viewed at wileyonlinelibrary.com]

To unravel the regulatory mechanisms governing gene expression of this group of six candidate genes, we conducted a detailed analysis of cis‐regulatory elements within the 2000 bp upstream regions of genes encoding these selected DEPs. Promoter analysis revealed a diverse array of cis‐regulatory elements, primarily involved in phytohormones and stress responses. Upon examining the percentage distribution across the three main categories (abiotic and biotic stress response, hormone response and plant development) for each candidate gene, intriguing patterns emerged (Figure 9a).

The two *HSPs* (17.4 and 18.2), *SYP121, PAL8* and *HDAC2*, displayed a balanced distribution of cis‐regulatory elements, with higher percentages in abiotic/biotic stress response (57.7%, 47%, 50%, 48.3% and 63.9%, respectively) and substantial contributions to hormone response (38.5%, 41.2%, 43.8%, 44.8% and 33.3%) (Table S4). Their promoters predominantly harbored MYB, MYC, LTR, anaerobic responsive element (ARE), drought inducible MYB‐binding sites (MBSs), osmotic stress‐responsive element (STRE) and wounding responsive elements belonging to the stress category, indicating a predominant involvement of these genes in abiotic more than biotic stress; additionally, ERE, abscisic acid responsiveness (ABRE), CGTCA‐ and TGACG‐motifs (involved in methyl‐jasmonate response) and AS‐1 (salicylic acid‐responsive element) were detected as the most abundant in hormone responsiveness element category. Notably, *Kat2* exhibited a predominant presence in the hormone response category (54.5%) with a lower distribution in the stress category (36.4%). Worth mentioning is that among all six analyzed candidates, *HDAC2* promoter harbors the highest number of cis‐elements related to the stress category (23 motifs) including ARE, MBS and MYC motifs strongly suggesting its involvement in anaerobic and oxidative stress response. However, a consistently low or absent percentage belonging to the plant development category was observed in all candidates, potentially highlighting the specialization of these genes in stress‐related pathways, rather than contributing extensively to developmental processes.

Having established the potential regulatory mechanism through cis‐element analysis, we analyzed the expression of this group of candidate genes in potato plants exposed to prolonged water deficit and salt treatment, until the onset of stress symptoms (Figure S7).

We investigated by qRT‐PCR their expression analysis, in 4‐week‐old potato plants subjected to 12 days of water withdrawal or exposed to increased salt concentration (up to 250 mM NaCl) for 1 week (Figure 9b,c).

Remarkably, alterations in transcript levels of all six candidate genes in treated potato plants perfectly mirrored changes in protein levels observed in adapted cells. Specifically, the gene encoding the downregulated PAL8 protein in adapted potato cells, also exhibited repression in plants as well as the five genes encoding the selected upregulated DEPs, were also significantly induced in plant response to both treatments (Figure 9c). In particular, the relative transcript levels of *HDAC2, HSP17.4* and *HSP18.2* were strongly up‐regulated approximately 9‐, 14‐ and 22‐fold respectively by prolonged water deficit, confirming their role as crucial drought‐responsive genes during adaptation. In response to salt treatment, the expression level of these genes slightly decreased, except for *HSP17.4* which was substantially up‐regulated by more than 30‐fold over the transcript level in control plants.

These findings collectively establish a correlation between regulatory mechanisms at the transcriptional level, as indicated by cis‐element analysis, and the transcript levels observed in plants exposed to drought and salt stress. This correlation not only supports the validity of our in vitro cellular model but also identifies a plethora of promising candidate genes linked to the potato's plant adaptive responses to osmotic stress.

## 4 Conclusions

This study focused on the molecular responses of potato cells to osmotic stress, examining both immediate and gradual impacts, and sheds light on the plants' adaptive mechanisms. By proteomic profiling, GO enrichment, KEGG pathway and MapMan analyses, we identified key proteins that are differentially expressed in response to the intensity and duration of stress. The identified proteins have a pivotal role in managing the stress conditions, in conserving energy‐ and preserving cellular functions, reflecting a multifaceted and dynamic adaptation mechanisms at the cellular level. Remarkably, we found that PEG‐adapted cells can tolerate various stress conditions, including anoxia, salt and heat stress. This implies the modulation of a conserved suite of stress response pathways, possibly involving the activation of master regulatory genes and signaling networks that confer a broad‐spectrum resilience and potentially describe a universal facet of plant stress responses. Further investigation into these conserved mechanisms could reveal key targets for genetic or biochemical modulation, paving the way for the development of crops with enhanced multi‐stress tolerance.

Our data reveal a proteomic signature that appears to be associated with plant cell adaptation. On the one hand, we documented a selective suppression of certain stress‐responsive proteins and pathways involved in amino acid and phenylpropanoid biosynthesis, as well as redox homeostasis in the adapted cells. In contrast, we simultaneously detected an enhanced expression of proteins involved in vital protective functions including chaperones and osmoprotectants, underscoring their critical role to tackle environmental challenges. Moreover, the identified sophisticated regulatory network, encompassing transcriptional, posttranscriptional, and epigenetic mechanisms likely orchestrates the global molecular changes that drive long‐term adaptation in plant cells. The quantification of selected metabolites and ROS provided a functional validation of our proteomic study.

Finally, when we extended our observations from cellular models to whole plants, we confirmed the expression patterns of a subset of genes encoding selected DEPs. This step demonstrates that our experimental model can provide fundamental insights into plant cell responses, supporting the broader applicability of our findings. Future studies will also focus on elucidating the biological roles these genes might have in adaptive processes, potentially opening new path in future breeding efforts to improve plant stress tolerance to water deficit.

Worth of note, despite the significant contributions of cellular models to fundamental research, it remains essential to acknowledge their inherent limitations when compared with whole‐organism models. While in vitro systems offer the advantage of controlled and reproducible conditions for simulating osmotic stress, they lack the complexity, and dynamic interactions present in whole plants. As an example, factors such as root‐shoot communication, tissue‐specific responses and plant‐microbiome are absent in cell cultures. Additionally, the absence of photosynthesis in the dark‐grown cells may result in significant metabolic differences when compared with plant tissues.

Additionally, efforts should focus on exploring the omics‐level variations between cell cultures and whole plants, to better contextualize these results and improve their relevance to whole plant physiology. Not least, future works on multi‐omics data integration and functional analyses will also ensure that interpretations of these data remain robust and contribute to a clearer understanding of potato adaptation mechanisms under water deficit conditions.

## Conflicts of Interest

The authors declare no conflicts of interest.

## Supporting information

Supporting information.

Supporting information.

Supporting information.

Supporting information.

Supporting information.

Supporting information.

## Data Availability

The data that support the findings of this study are openly available in ProteomeXchange Consortium at https://www.proteomexchange.org, reference number PXD050665.
