# Supplementary material for: Proteome Reprogramming and Acquired Stress Tolerance in Potato Cells Exposed to Acute or Stepwise Water Deficit
Source: Plant Cell Environ. 2024 Dec 5;48(5):2875–94. doi: 10.1111/pce.15306 (PMC11963495; doi:10.1111/pce.15306)
Supplement: Supplementary file 1 — Supporting information. [file PCE-48-2875-s003.docx]

**Proteome reprogramming and acquired stress tolerance in potato cells exposed to acute or stepwise water deficit**

Elisa Cappetta^1^*, Carmine Del Regno^1,2^, Sara Ceccacci^1,3^, Maria Chiara Monti^1,4^, Lucio Spinelli^4^, Marisa Conte^1^, Chiara D’Anna^1^, Mariaevelina Alfieri^5^, Mariapia Vietri^1^, Antonello Costa^6^, Antonietta Leone^1^, Alfredo Ambrosone^1^*

^1^ Department of Pharmacy, University of Salerno, 84084, Fisciano, Italy

^2^ SAFE - School of Agricultural, Forest, Food, and Environmental Sciences, University of Basilicata, 10, Viale dell'Ateneo Lucano - Potenza (PZ), Italy

^3^ Proteomics Platform Necker, Université Paris Cité-Structure Fédérative de Recherche Necker, INSERM US24/CNRS UAR3633, 75015, Paris, France

^4^ Department of Pharmacy, University of Naples 'Federico II', Via Tommaso De Amicis 95, 80131 Naples, Italy

^5^ Clinical Pathology, Santobono-Pausilipon Children’s Hospital, AORN, 80122 Naples, Italy.

^6^ National Research Council of Italy, Institute of Biosciences and BioResources, Research Division Portici (CNR-IBBR), 80055, Italy

* Corresponding authors: Elisa Cappetta ([ecappetta@unisa.it](mailto:ecappetta@unisa.it)); Alfredo Ambrosone ([aambrosone@unisa.it](mailto:aambrosone@unisa.it))

| Table S1. List of primers used in this study. | | |  |  |  |
| --- | --- | --- | --- | --- | --- |
| **Gene** | **Description** | **Ref seq** | | **Forward primer** | **Reverse primer** |
| *HSP17.4* | 17.4 kDa class III heat shock protein | XM_006342843.2 | | GTAGCATTCCCGTGGACATT | CTTCACTCTCCTCACGCTTC |
| *HSP18.2* | 18.2 kDa class I heat shock protein-like | XM_006345187.2 | | GATCCACTCGAAGGTTTCCC | CTTCCGGGGTTTCTTTCCAA |
| *SYP121* | syntaxin-121-like | XM_006339110.2 | | GGAGCAAGGAAGAGGACAAG | TGTGCTCCTTGACTTTCCAC |
| *Kat2* | 3-ketoacyl-CoA thiolase 2, peroxisomal | XM_006360270.2 | | TGGTGCTGGATTGGAGTCTA | TCTTGTTCCTGCCTTGTCAC |
| *PAL8* | phenylalanine ammonia-lyase-like | XM_006345724.2 | | AGGTGCTGAAATCGCTATGG | ACTGCCTCCTCTGTCTTTCT |
| *HDAC2* | Histone Deacetylase | XM_015309972.1 | | GAGCAGAAGAAGAGACCAGC | TTTGGGGACTGAGGTTTTGG |
| *ACTIN* | Actin | NM_001288158.1 | | GGACTCTGGTGATGGTGTCA | GTGAACGAGTAACCACGCTC |


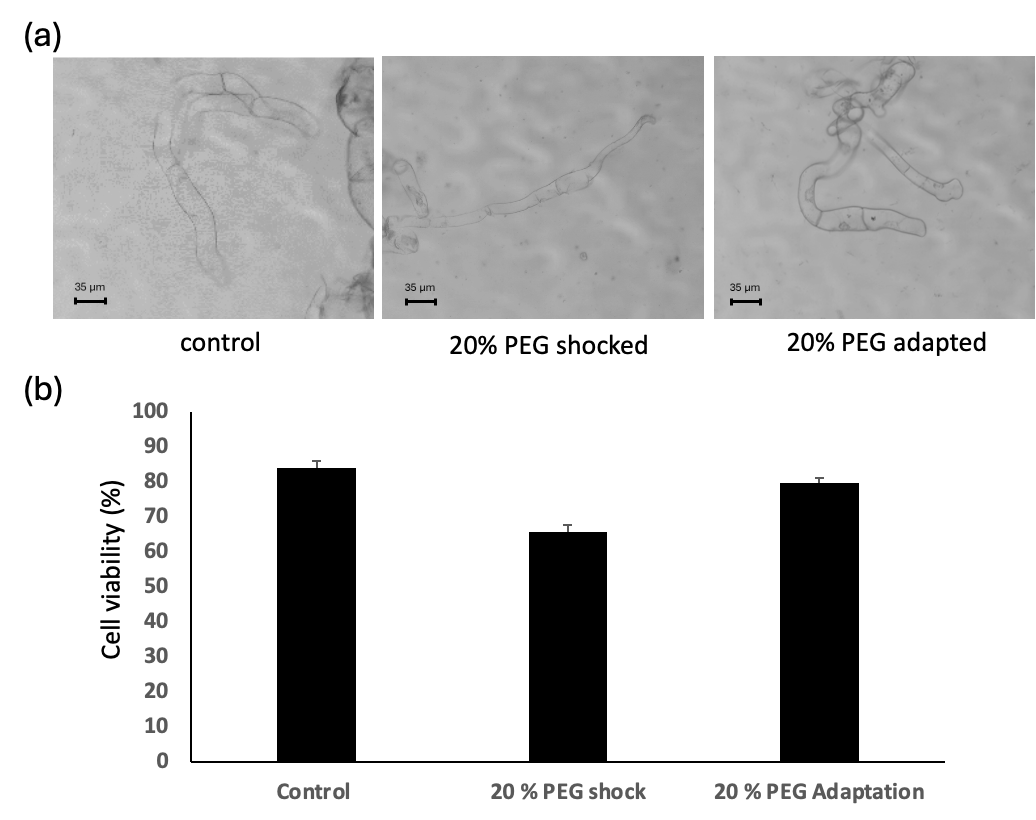


**Figure S1:** Effect of osmotic stress on cell morphology and viability. (a) Microscopic images of cells in control, 20% shocked and 20% PEG adapted conditions. (b) A bar graph representing cell viability (%) for the same three conditions estimated by using Fluorescein diacetate.

**Figure S2:** Panel A shows the box-plot of the proteins abundance of each PEG treated and untreated sample. Panel B shows the box-plot of the proteins abundance of grouped PEG treated and untreated samples.

**Figure S3:** Principal component analysis (PCA) plot is reported using the Scores Plot. Circles represent grouped samples treated and untreated with PEG. Both the x axis and y axis displays principal components together with their percentage values.

**Table S2.** Kegg pathway and corresponding number of proteins for the four analyzed conditions

| **Pathway** | **10% PEG stress down** | **20% PEG stress down** | **10% PEG adapted down** | **20% PEG adapted down** | **10% PEG stress up** | **20% PEG stress up** | **10% PEG adapted up** | **20% PEG adapted up** |
| --- | --- | --- | --- | --- | --- | --- | --- | --- |
| 2-Oxocarboxylic acid metabolism | 0 | 0 | 5 | 0 | 0 | 0 | 0 | 0 |
| Alanine aspartate and glutamate metabolism | 0 | 0 | 0 | 0 | 2 | 0 | 0 | 0 |
| Amino sugar and nucleotide sugar metabolism | 0 | 0 | 0 | 0 | 0 | 2 | 0 | 0 |
| Arginine and proline metabolism | 0 | 0 | 0 | 0 | 2 | 0 | 0 | 0 |
| Arginine biosynthesis | 0 | 0 | 0 | 0 | 2 | 0 | 0 | 0 |
| Ascorbate and aldarate metabolism | 0 | 0 | 0 | 0 | 2 | 0 | 0 | 0 |
| Beta-Alanine metabolism | 0 | 0 | 0 | 0 | 0 | 2 | 0 | 0 |
| Biosynthesis of amino acids | 0 | 0 | 9 | 0 | 3 | 0 | 0 | 0 |
| Biosynthesis of cofactors | 0 | 0 | 4 | 0 | 3 | 0 | 0 | 0 |
| Biosynthesis of unsaturated fatty acids | 0 | 0 | 0 | 0 | 0 | 0 | 0 | 2 |
| Biotin metabolism | 0 | 0 | 4 | 0 | 0 | 0 | 0 | 0 |
| Butanoate metabolism | 0 | 0 | 3 | 0 | 0 | 0 | 0 | 0 |
| C5-Branched dibasic acid metabolism | 0 | 0 | 3 | 0 | 0 | 0 | 0 | 0 |
| Carbon fixation in photosynthetic organisms | 0 | 0 | 0 | 0 | 3 | 0 | 0 | 0 |
| Carbon metabolism | 0 | 0 | 5 | 0 | 7 | 6 | 0 | 0 |
| Citrate cycle (TCA cycle) | 0 | 0 | 0 | 0 | 2 | 3 | 0 | 0 |
| Cysteine and methionine metabolism | 0 | 0 | 0 | 0 | 2 | 0 | 0 | 0 |
| Endocytosis | 0 | 0 | 0 | 5 | 0 | 0 | 0 | 0 |
| Fatty acid biosynthesis | 0 | 0 | 4 | 0 | 0 | 0 | 0 | 0 |
| Fatty acid metabolism | 0 | 0 | 4 | 0 | 0 | 0 | 0 | 0 |
| Galactose metabolism | 0 | 0 | 2 | 0 | 0 | 0 | 0 | 0 |
| Glutathione metabolism | 0 | 0 | 0 | 6 | 2 | 0 | 0 | 0 |
| Glycine serine and threonine metabolism | 0 | 0 | 2 | 0 | 0 | 0 | 0 | 0 |
| Glycolysis/Gluconeogenesis | 0 | 0 | 3 | 0 | 0 | 2 | 0 | 0 |
| Glyoxylate and dicarboxylate metabolism | 0 | 0 | 0 | 0 | 2 | 2 | 0 | 0 |
| Histidine metabolism | 0 | 0 | 0 | 0 | 0 | 1 | 0 | 0 |
| Inositol phosphate metabolism | 2 | 0 | 0 | 0 | 0 | 0 | 0 | 0 |
| Limonene and pinene degradation | 0 | 0 | 0 | 0 | 0 | 1 | 0 | 0 |
| Nitrogen metabolism | 0 | 0 | 0 | 0 | 2 | 0 | 0 | 0 |
| Nucleotide excision repair | 0 | 0 | 0 | 0 | 0 | 0 | 2 | 0 |
| Nucleotide metabolism | 0 | 2 | 0 | 0 | 0 | 0 | 0 | 0 |
| Pantothenate and CoA biosynthesis | 0 | 0 | 2 | 0 | 0 | 0 | 0 | 0 |
| Pentose and glucuronate interconversions | 0 | 0 | 0 | 5 | 0 | 0 | 0 | 0 |
| Pentose phosphate pathway | 0 | 0 | 2 | 0 | 0 | 0 | 0 | 0 |
| Phenylalanine metabolism | 0 | 0 | 0 | 0 | 3 | 3 | 0 | 0 |
| Phenylalanine tyrosine and tryptophan biosynthesis | 0 | 0 | 2 | 0 | 2 | 0 | 0 | 0 |
| Phenylpropanoid biosynthesis | 0 | 0 | 6 | 11 | 6 | 5 | 0 | 0 |
| Propanoate metabolism | 0 | 0 | 0 | 0 | 0 | 2 | 0 | 0 |
| Proteasome | 3 | 0 | 2 | 0 | 0 | 0 | 0 | 0 |
| Protein processing in endoplasmic reticulum | 0 | 0 | 0 | 0 | 0 | 0 | 0 | 9 |
| Purine metabolism | 0 | 2 | 0 | 0 | 0 | 0 | 0 | 0 |
| Pyruvate metabolism | 0 | 0 | 0 | 0 | 0 | 2 | 0 | 0 |
| Riboflavin metabolism | 0 | 0 | 0 | 0 | 0 | 0 | 2 | 0 |
| Ribosome | 5 | 3 | 0 | 0 | 0 | 0 | 0 | 0 |
| Spliceosome | 0 | 4 | 0 | 0 | 0 | 0 | 0 | 0 |
| Tryptophan metabolism | 0 | 0 | 0 | 0 | 2 | 2 | 0 | 0 |
| Valine leucine and isoleucine biosynthesis | 0 | 0 | 3 | 0 | 2 | 3 | 0 | 0 |
| Viral life cycle-HIV-1 | 0 | 2 | 0 | 0 | 0 | 0 | 0 | 0 |

|  |  | | |  | | |  | |  |
| --- | --- | --- | --- | --- | --- | --- | --- | --- | --- |
|  | **Table S3.** ID and description of proteins belonging the most enriched KEGG pathways | | | | | | |  |  |
| **Pathway** | **Proteins** | | | | |  |  |  |  |
|  | **10% PEG STRESS UP** | **20% PEG STRESS UP** | **10% PEG ADAPTED DOWN** | | **20% PEG ADAPTED DOWN** |  |  |  |  |
| **Phenylalanine metabolism** | M1C5K7 (Phenylalanine ammonia-lyase) | M1C5K7 (Phenylalanine ammonia-lyase) |  | |  |  |  |  |  |
|  | M1BY02 (Phenylalanine ammonia-lyase) | M1BY02 (Phenylalanine ammonia-lyase) |  | |  |  |  |  |  |
|  | M1BTN1 (Aspartate aminotransferase ) | M1CHL0 (Aromatic amino acid decarboxylase 2 ) |  | |  |  |  |  |  |
|  |  |  |  | |  |  |  |  |  |
| **Phenylpropanoid biosynthesis** | M1C3C2 (Peroxidase) | M1AU65 (Peroxidase) | M1CE55 (Peroxidase) | | M0ZIC4 (Peroxidase) |  |  |  |  |
|  | M1AU65 (Peroxidase) | M1C5K7 (Phenylalanine ammonia-lyase ) | M1C3C2 (Peroxidase | | M1CE55 (Peroxidase) |  |  |  |  |
|  | M1C5K7 (Phenylalanine ammonia-lyase ) | M1AY17 (Peoxidase) | M1B986 (Peroxidase) | | M1C3C2 (Peroxidase) |  |  |  |  |
|  | M1BY02 (Phenylalanine ammonia-lyase ) | M1BY02 (Phenylalanine ammonia-lyase ) | M1B2E4 (Peroxidase) | | M1C5K7 (Phenylalanine ammonia-lyase ) |  |  |  |  |
|  | M1B3Q2 (Peroxidase) | M1B3Q2 (Peroxidase) | M1C183 (Peroxidase) | | M1AY17 (Peroxidase) |  |  |  |  |
|  | M1B2E4 (Perosxidase) |  | M1AP83 (Peroxidase) | | M1C911 (Peroxidase) |  |  |  |  |
|  |  |  |  | | M1B986 (Peroxidase) |  |  |  |  |
|  |  |  |  | | M1A5B5 (Peroxidase) |  |  |  |  |
|  |  |  |  | | M1ASL1 (Catechol O-methyltransferase ) |  |  |  |  |
|  |  |  |  | | M1B2E4 (Peroxidase) |  |  |  |  |
|  |  |  |  | | M1AP83 (Peroxidase) |  |  |  |  |
|  |  |  |  | |  |  |  |  |  |
| **Carbon metabolism** | M1AFA9 (Glutamate dehydrogenase ) | M1BSG4 (Succinate--CoA ligase [ADP-forming] subunit beta, mitochondrial | Q8VWX3 (Phosphotransferase ) | |  |  |  |  |  |
|  | M1BB73 (Glutamate dehydrogenase ) | M1CNJ1 (oxoglutarate dehydrogenase (succinyl-transferring) | M1AP15 (Glycine cleavage system H protein ) | |  |  |  |  |  |
|  | M1BPR5 (ribose-5-phosphate isomerase ) | M1AQ24 (methylmalonate-semialdehyde dehydrogenase (CoA acylating) ) | M1BQ10 (ribulose-phosphate 3-epimerase ) | |  |  |  |  |  |
|  | M1C5X7 (oxoglutarate dehydrogenase (succinyl-transferring) | M1C5X7 (oxoglutarate dehydrogenase (succinyl-transferring) | M1BT61 (Succinic semialdehyde reductase isofom1) | |  |  |  |  |  |
|  | M1BTN1 (Aspartate aminotransferase ) | M1CL86 (Dihydrolipoyl dehydrogenase ) | M1CF07 (Pyruvate kinase) | |  |  |  |  |  |
|  | M1CL86 (Dihydrolipoyl dehydrogenase ) | M1B8S4 (Malate dehydrogenase) |  | |  |  |  |  |  |
|  | M1B8S4 (Malate dehydrogenase) |  |  | |  |  |  |  |  |
|  |  |  |  | |  |  |  |  |  |
| **TCA** | M1C5X7 (oxoglutarate dehydrogenase (succinyl-transferring) | M1CNJ1 (oxoglutarate dehydrogenase (succinyl-transferring) |  | |  |  |  |  |  |
|  | M1CL86 Dihydrolipoyl dehydrogenase | M1C5X7 (oxoglutarate dehydrogenase (succinyl-transferring) |  | |  |  |  |  |  |
|  |  | M1CL86 Dihydrolipoyl dehydrogenase |  | |  |  |  |  |  |

| DEPs in shock conditions 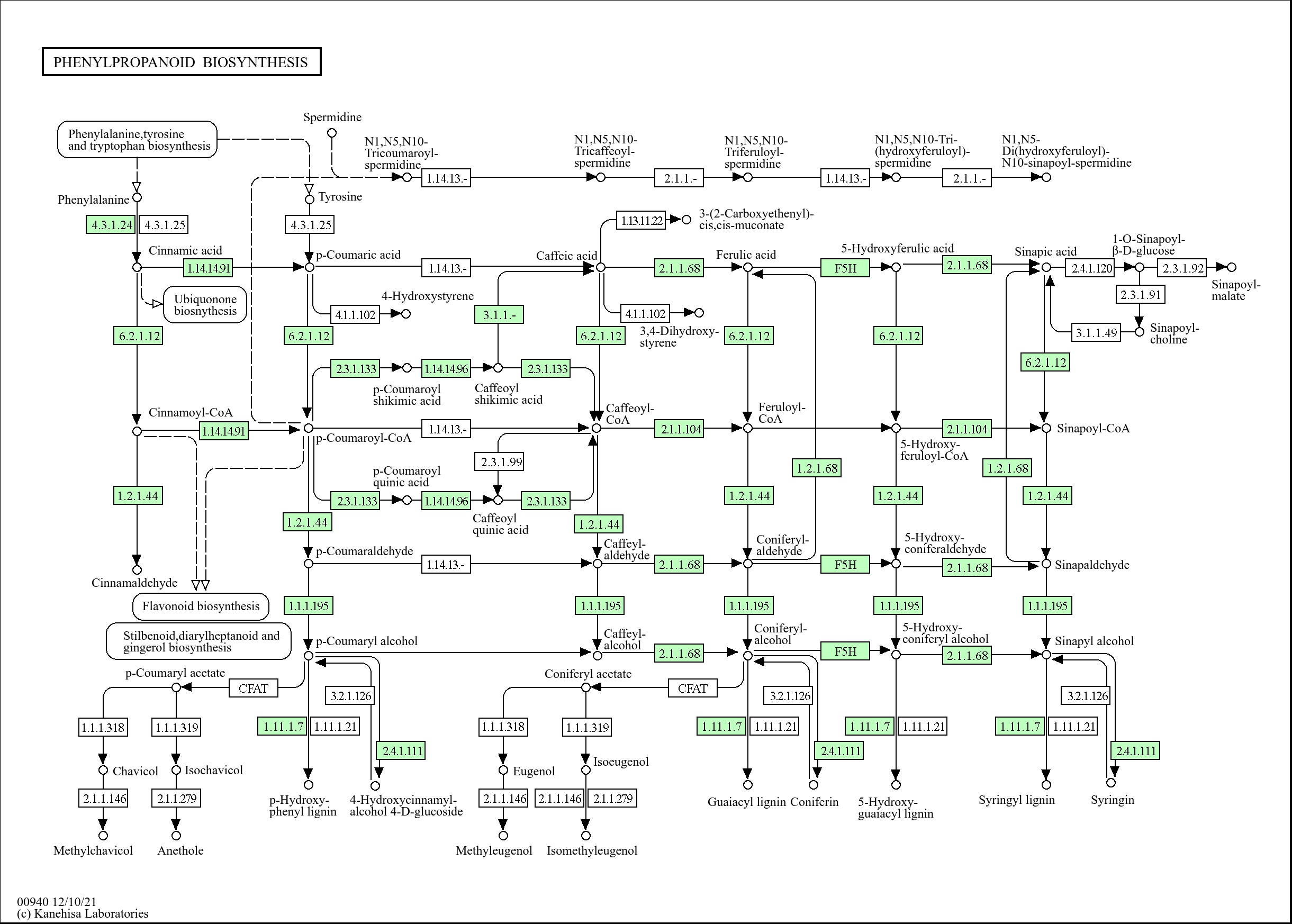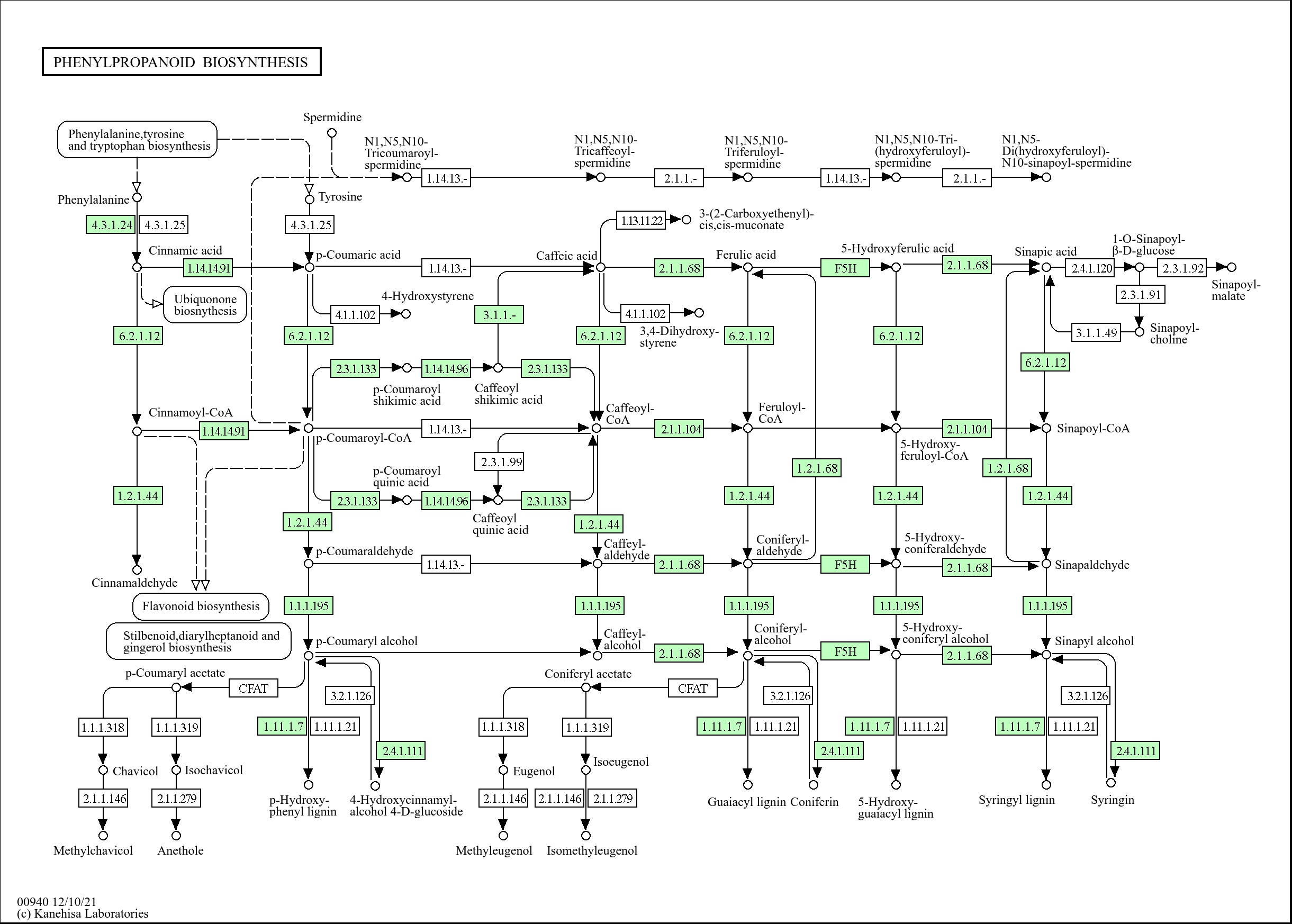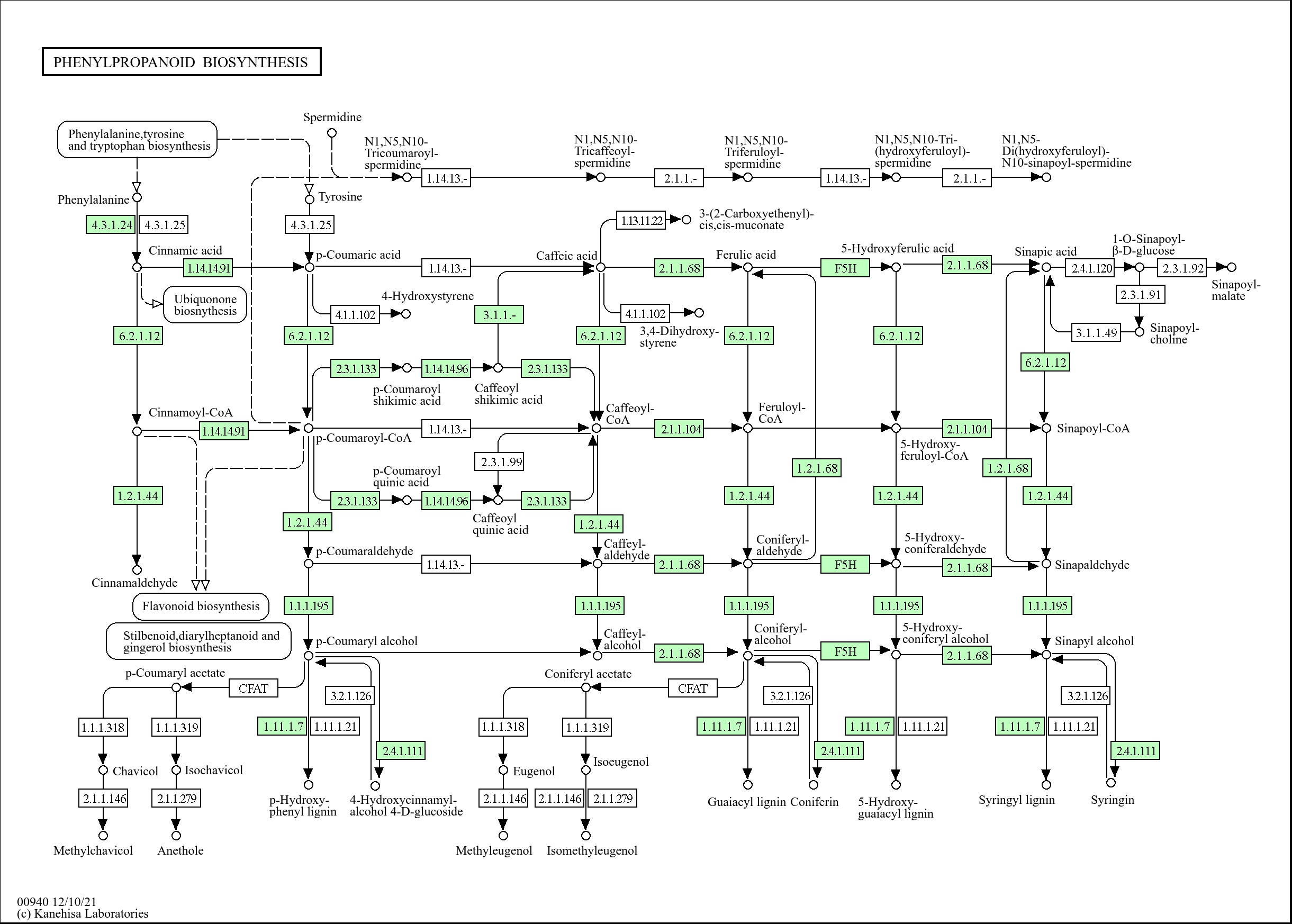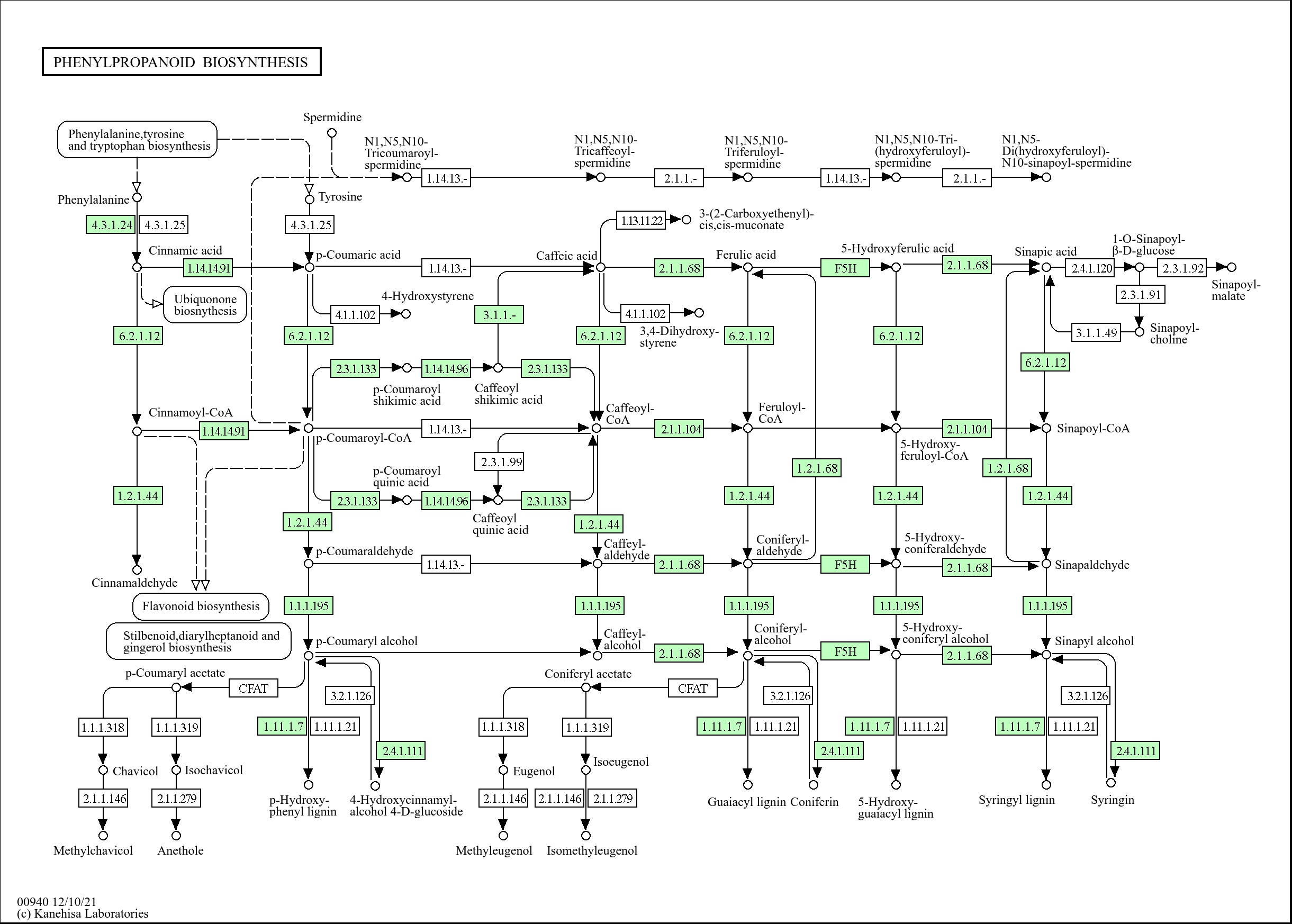 10% PEG (moderate condition)  20% PEG (severe condition)  10% PEG (moderate condition)  20% PEG (severe condition)  DEPs in adapted conditions  (a)  (b)  **Figure S4.** DEPs involved in the phenylpropanoid KEGG pathway in shock (a) and adapted (b) conditions. Upregulated enriched proteins are indicated by a red background. Downregulated enriched proteins are indicated by a blue background. 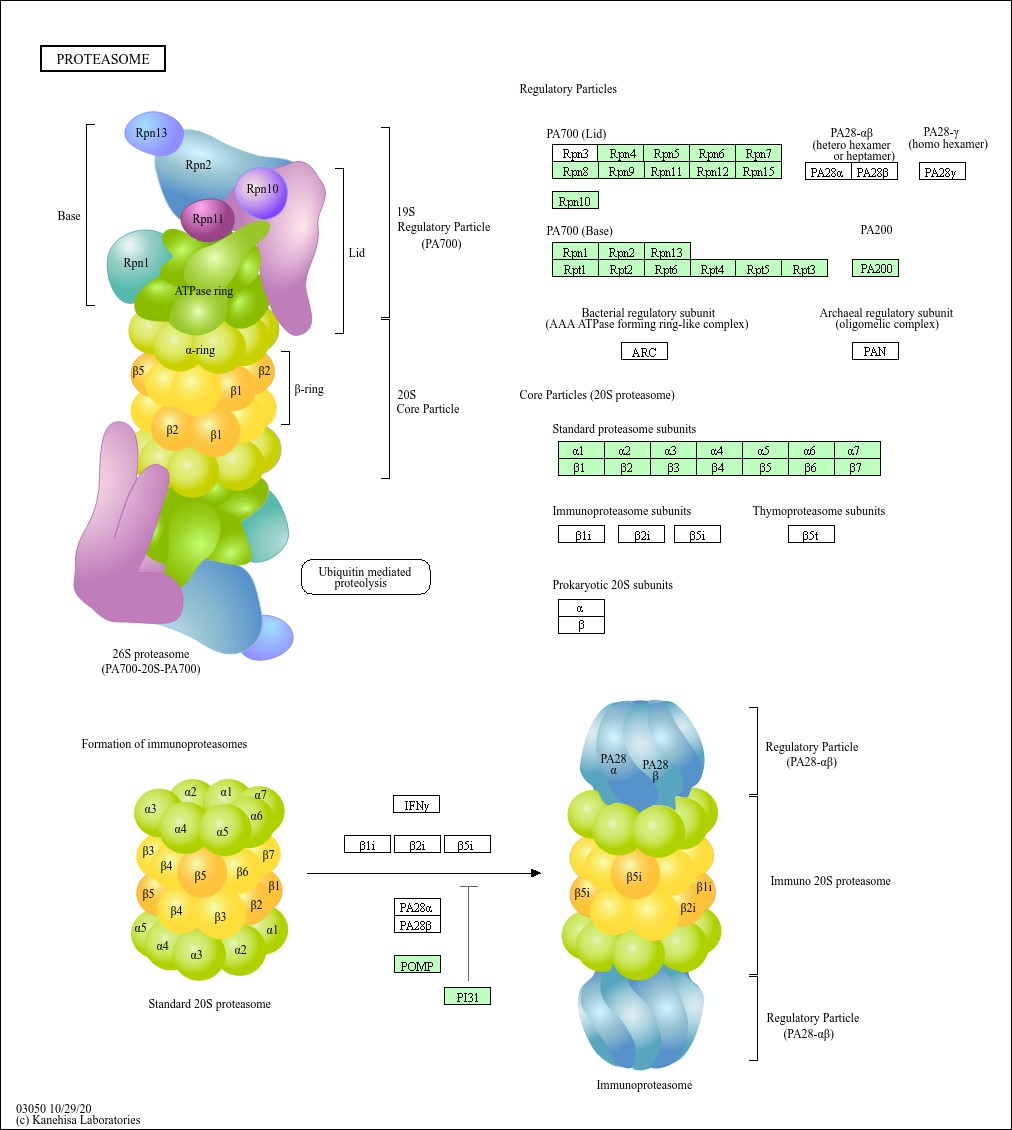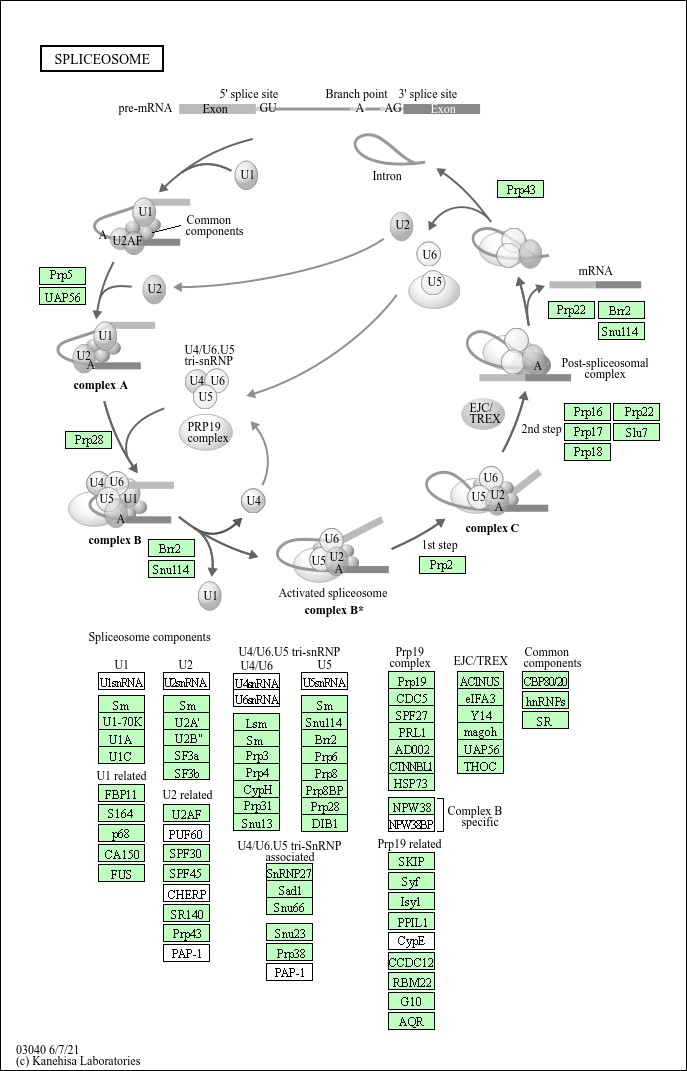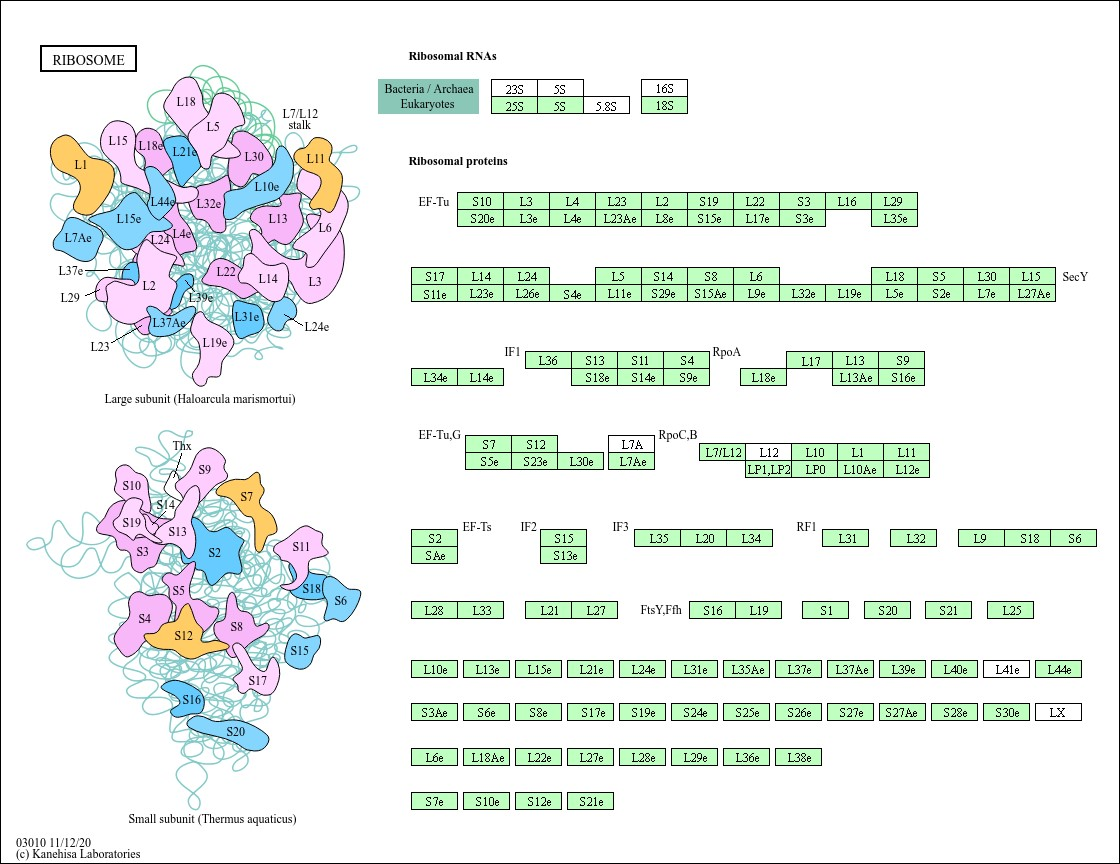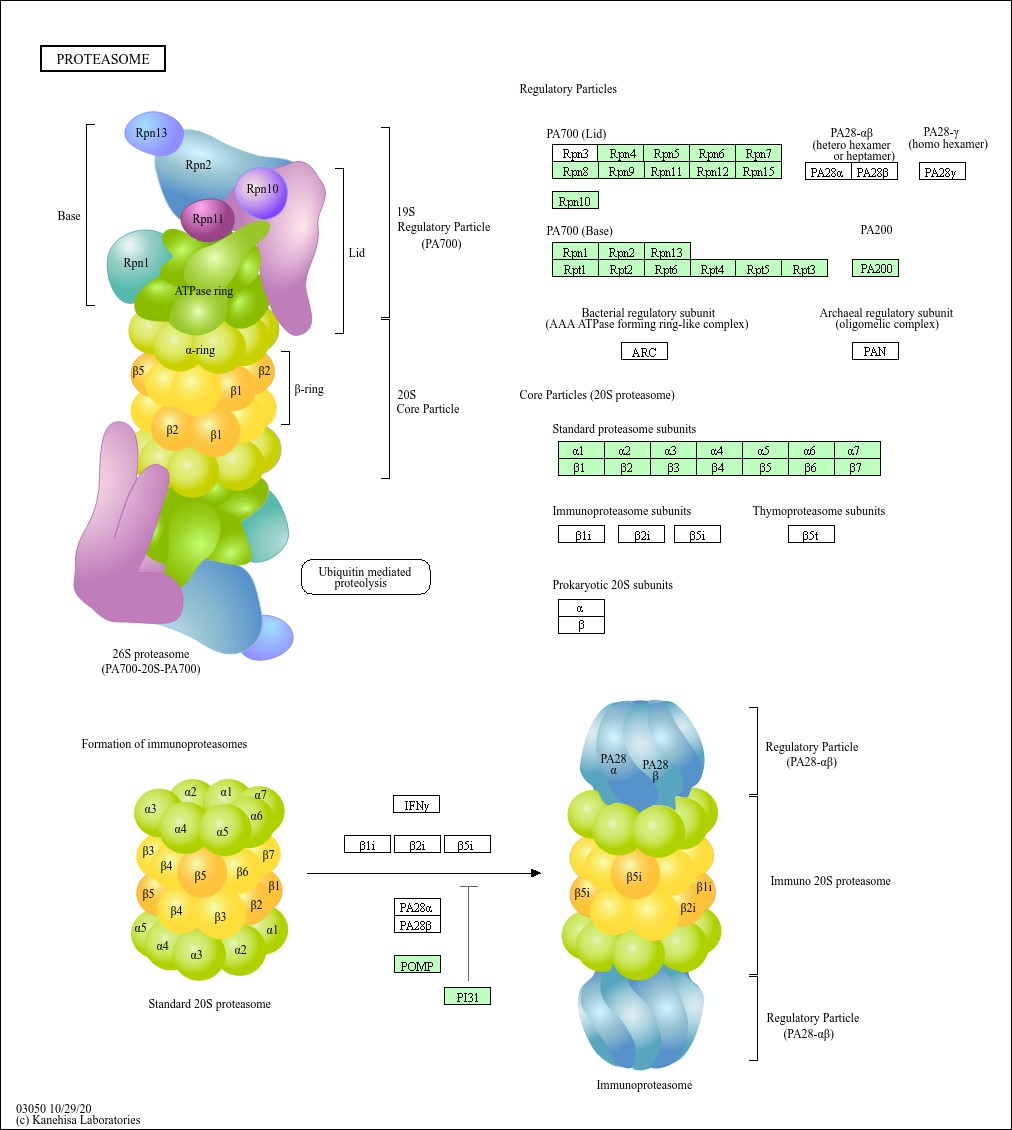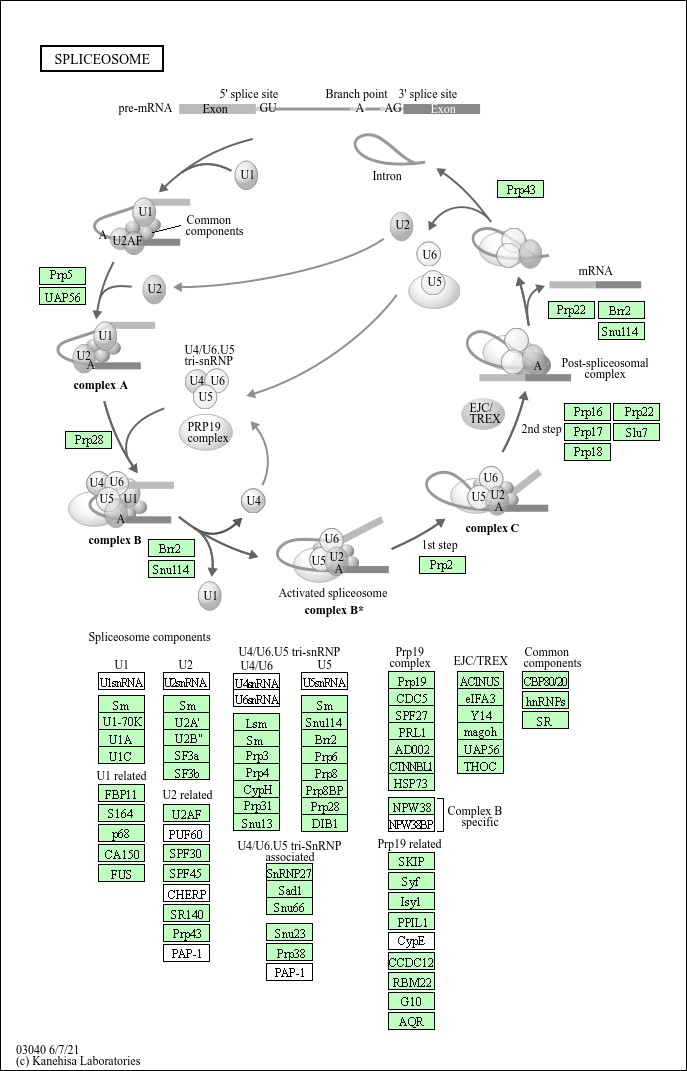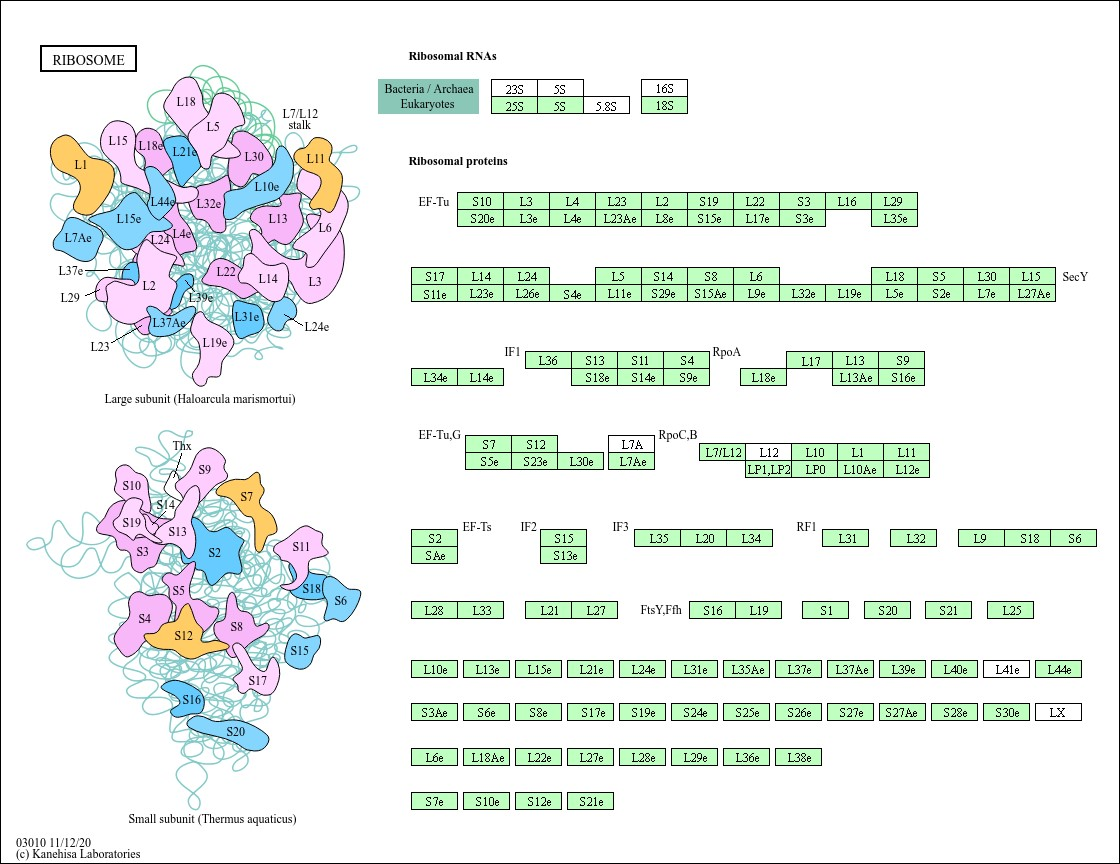 Downregulated proteins in 10% shocked cells  Downregulated proteins in 20% shocked cells  (a)  (b)  **Figure S5**. Downregulated proteins involved in the ribosome, spliceosome and proteosome KEGG pathway in 10% (a) and 20% (b) shocked cells. Proteins enriched are indicated by a blue background.  **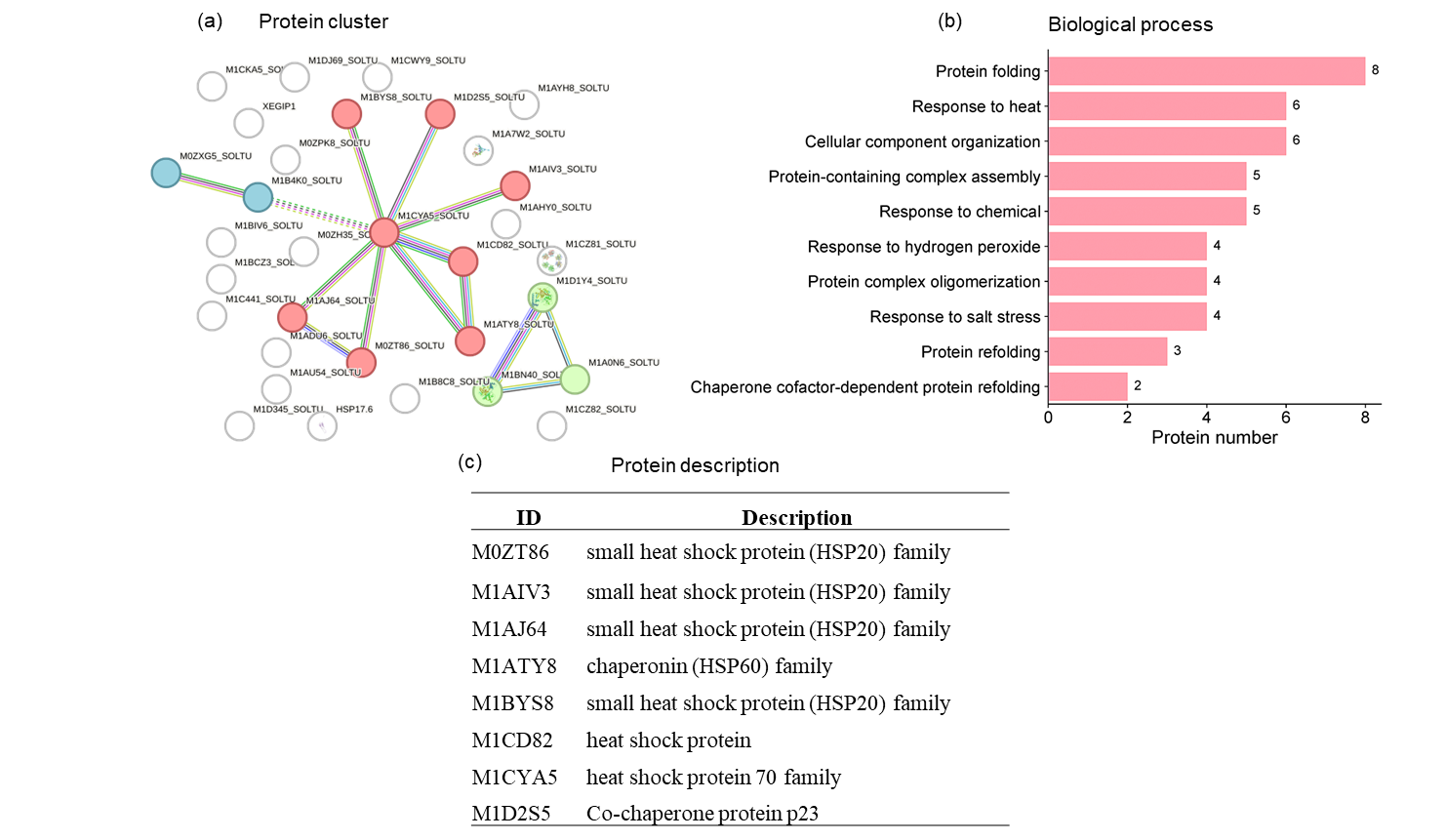**  **Figure S6.** The protein-protein interaction (PPI) network analysis of upregulated DEPs in 20% PEG adapted cells belonging BIN 19. (a) STRING interaction analysis of 24 upregulated DEPs was reported. The cluster in the network of upregulated proteins was identified using the MCL clustering algorithm and colored in pink. (b) Histogram representing Gene Ontology (biological process) terms for the identified cluster. (c) Description of 8 proteins identified in the cluster.  **Table S4.** List of cis-elements identified analyzing promoter region of the selected genes. The distribution across the three main categories (abiotic and biotic stress response, hormone response, and plant development) was reported. | | | | | | | | | | | | | | | | | | | | |  |
| --- | --- | --- | --- | --- | --- | --- | --- | --- | --- | --- | --- | --- | --- | --- | --- | --- | --- | --- | --- | --- | --- |
|  | |  | | | | | | | |  | | | | | | | |  | | |  |
|  | | **GENE NAME** | **AP-1** | **ARE** | **BOX-S** | **CCAAT-box** | **LTR** | **MBS** | **MYB** | **MYB-like sequence** | | **MYB-recognition site** | **MYC** | **STRE** | **W box** | **WRE3** | **WUN-motif** |  |  |  |  |
|  | | HSP 17.4 | 0 | 1 | 0 | 0 | 3 | 1 | 4 | 0 | | 0 | 1 | 2 | 0 | 1 | 2 |  |  |  |  |
|  |  | HSP18.2 | 4 | 0 | 0 | 0 | 0 | 1 | 0 | 0 | | 0 |  | 0 | 1 | 1 | 1 |  |  |  |  |
|  |  | SYP121 | 0 | 2 | 0 | 1 | 0 | 0 | 6 | 3 | | 1 | 0 | 1 | 1 | 1 | 0 |  |  |  |  |
|  |  | Kat2 | 0 | 2 | 0 | 0 | 0 | 2 | 2 | 0 | | 0 | 0 | 1 | 0 | 0 | 1 |  |  |  |  |
|  |  | PAL8 | 0 | 1 | 0 | 2 | 0 | 0 | 6 | 0 | | 2 | 0 | 1 | 2 | 0 | 0 |  |  |  |  |
|  |  | HDAC2 | 1 | 4 | 1 | 0 | 0 | 2 | 9 | 2 | | 0 | 2 | 2 | 0 | 0 | 0 |  |  |  |  |
|  | | **GENE NAME** | **ABRE** | **ABRE2** | **ABRE3a** | **ABRE4** | **AS-1** | **CGTCA-motif** | **ERE** | **O2-site** | | **P-box** | **TATC-box** | **TCA** | **TCA-element** | **TGACG-motif** | **TGA-element** | | |  |  |
|  | | HSP 17.4 | 1 | 1 | 0 | 1 | 2 | 2 | 0 | 0 | | 1 | 0 | 0 | 0 | 2 | 0 | | |  |  |
|  |  | HSP18.2 | 0 | 0 | 0 | 0 | 1 | 1 | 2 | 1 | | 0 | 0 | 0 | 0 | 1 | 1 | | |  |  |
|  |  | SYP121 | 2 | 2 | 0 | 2 | 2 | 2 | 0 | 0 | | 1 | 1 | 0 | 0 | 2 | 0 | | |  |  |
|  |  | Kat2 | 2 | 1 | 0 | 1 | 1 | 1 | 3 | 1 | | 0 | 0 | 0 | 1 | 1 | 0 | | |  |  |
|  |  | PAL8 | 3 | 0 | 0 | 0 | 1 | 1 | 5 | 0 | | 1 | 0 | 0 | 1 | 1 | 0 | | |  |  |
|  |  | HDAC2 | 2 | 0 | 0 | 0 | 2 | 2 | 2 | 0 | | 0 | 0 | 0 | 1 | 2 | 1 | | |  |  |
|  | | **GENE NAME** | **BOX III** | **CAT-box** | **GCN4-motif** | **MBSI** | **MSA-like** |  |  |  | |  |  |  |  |  |  |  |  |  |  |
|  | | HSP 17.4 | 1 | 0 | 0 | 0 | 0 |  |  |  | |  |  |  |  |  |  |  |  |  |  |
|  |  | HSP18.2 | 0 | 0 | 2 | 0 | 0 |  |  |  | |  |  |  |  |  |  |  |  |  |  |
|  |  | SYP121 | 0 | 1 | 1 | 0 | 0 |  |  |  | |  |  |  |  |  |  |  |  |  |  |
|  |  | Kat2 | 1 | 0 | 0 | 1 | 0 |  |  |  | |  |  |  |  |  |  |  |  |  |  |
|  |  | PAL8 | 0 | 0 | 1 | 0 | 1 |  |  |  | |  |  |  |  |  |  |  |  |  |  |
|  |  | HDAC2 | 0 | 0 | 1 | 0 | 0 |  |  |  | |  |  |  |  |  |  |  |  |  |  |


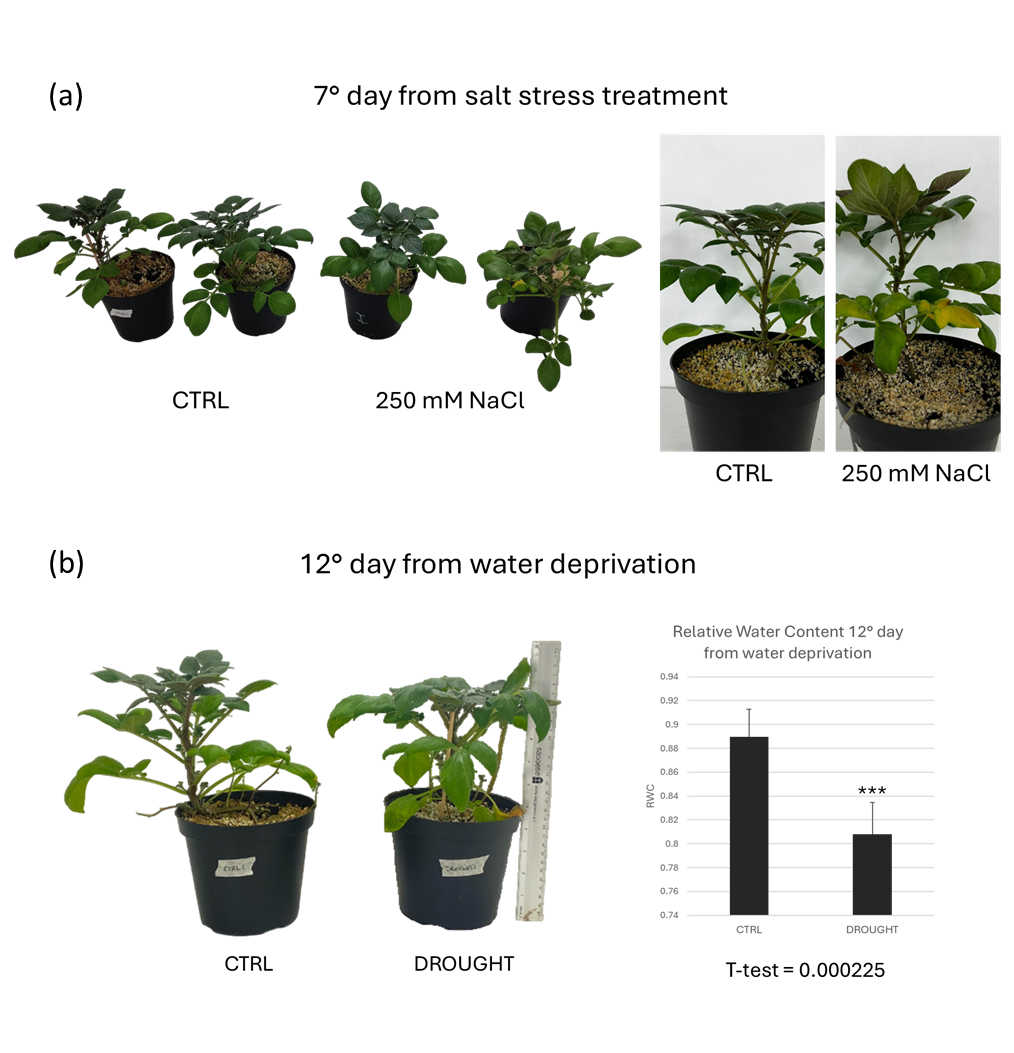


**Figure S7.** Stress condition assessment in potato plants. (a) salt stress visual damage on the 7^th^ day from the first saline treatment. (b) visual loss of leaves turgidity and relative water content on the 12^th^ day from water deprivation.
